# Progesterone receptor membrane associated component 1 enhances obesity progression in mice by facilitating lipid accumulation in adipocytes

**DOI:** 10.1038/s42003-020-01202-x

**Published:** 2020-09-04

**Authors:** Ryogo Furuhata, Yasuaki Kabe, Ayaka Kanai, Yuki Sugiura, Hitoshi Tsugawa, Eiji Sugiyama, Miwa Hirai, Takehiro Yamamoto, Ikko Koike, Noritada Yoshikawa, Hirotoshi Tanaka, Masahiro Koseki, Jun Nakae, Morio Matsumoto, Masaya Nakamura, Makoto Suematsu

**Affiliations:** 1 Department of Biochemistry, Keio University School of Medicine, 35 Shinanomachi, Shinjuku-ku, Tokyo 160-8582 Japan; 2 Department of Orthopaedic[s] Surgery, Keio University School of Medicine, 35 Shinanomachi, Shinjuku-ku, Tokyo 160-8582 Japan; 3 Japan Agency for Medical Research and Development, Core Research for Evolutional Science and Technology (AMED-CREST), Tokyo, Japan; 4 Department of Rheumatology and Allergy, IMSUT Hospital, The Institute of Medical Science, The University of Tokyo, Tokyo, Japan; 5 Department of Cardiovascular Medicine, Osaka University Graduate School of Medicine, Suita, Osaka 565-0871 Japan; 6 Department of Physiology, International University of Health and Welfare School of Medicine, Narita, 286-8686 Japan

**Keywords:** Lipids, Protein transport, Fat metabolism, Metabolic diseases

## Abstract

Progesterone receptor membrane associated component 1 (PGRMC1) exhibits haem-dependent dimerization on cell membrane and binds to EGF receptor and cytochromes P450 to regulate cancer proliferation and chemoresistance. However, its physiological functions remain unknown. Herein, we demonstrate that PGRMC1 is required for adipogenesis, and its expression is significantly enhanced by insulin or thiazolidine, an agonist for PPARγ. The haem-dimerized PGRMC1 interacts with low-density lipoprotein receptors (VLDL-R and LDL-R) or GLUT4 to regulate their translocation to the plasma membrane, facilitating lipid uptake and accumulation, and de-novo fatty acid synthesis in adipocytes. These events are cancelled by CO through interfering with PGRMC1 dimerization. PGRMC1 expression in mouse adipose tissues is enhanced during obesity induced by a high fat diet. Furthermore, adipose tissue-specific PGRMC1 knockout in mice dramatically suppressed high-fat-diet induced adipocyte hypertrophy. Our results indicate a pivotal role of PGRMC1 in developing obesity through its metabolic regulation of lipids and carbohydrates in adipocytes.

## Abstract (web summary)

Ryogo Furuhata, Yasuaki Kabe et al. discover that PGRMC1 is required for adipogenesis in mice and its expression is increased by insulin or PPARγ agonists. They find that knocking out *PGRMC1* protects against diet-induced obesity by regulating lipid uptake and accumulation.

## Introduction

Globally, metabolic syndrome is one of the most serious clinical manifestations, which is characterized by several lines of metabolic dysfunction, including obesity, hyperglycemia, hyperlipidemia, and hypertension. Excess intake of diets containing carbohydrates and fats leads to adipocyte hypertrophy and fat expansion, resulting in obesity^1,2^. White adipose tissue (WAT) stores excess energy-generating substrates in the form of triglycerides (TG) in cellular lipid droplets.

Adipocytes store lipids inside the cells and hypertrophy of adipocytes is induced by excess metabolic energy^3^. Adipogenesis is induced in response to adipogenic inducers such as insulin, cyclic adenosine monophosphate (cAMP), and dexamethasone through mechanisms involving adipogenic transcription factors such as CCAAT/enhancer-binding protein (C/EBP) gene family and peroxisome proliferator-activated receptor gamma (PPARγ)^4^. PPARγ induces lipid accumulation in adipocytes by transactivating the genes involved in the transport of fatty acids such as fatty acid-binding protein 4 (FABP4) or CD36^5^, or in insulin signaling and glucose uptake regulators, such as insulin receptor substrates 2^6^ or glucose transporter 4 (GLUT4)^7,8^. Hyperglycemia induces insulin signaling that promotes translocation of GLUT4 from intracellular vesicles to the plasma membrane via activation of its downstream pathway, phosphatidylinositol 3 kinase/protein kinase B (PI3K/Akt), resulting in the facilitation of glucose uptake into adipocytes^9,10^. The incorporated glucose is utilized for de novo fatty acid synthesis. In addition, the elevation of glucose or FFA in blood also promotes secretion of triglyceride (TG)-rich lipoproteins in the liver, leading to an increase in low-density lipoproteins (LDL) or very-low-density lipoproteins (VLDL) into circulation. Plasma LDL or VLDL are incorporated into the adipocyte cells mediated by LDL receptor (LDL-R) or VLDL receptor (VLDL-R), via endocytosis^11–13^. Although adipocytes accumulate lipids inside the cells by incorporating glucose or lipids, fundamental molecular mechanisms underlying the activation of adipogenic stimulators for incorporating and accumulating energy resources in adipocytes remain largely unknown.

Progesterone receptor membrane-associated component 1 (PGRMC1) is a haem-binding membrane protein that belongs to the membrane-associated progesterone receptor protein family^14^. PGRMC1 is highly expressed in a wide variety of solid cancers^15–18^ and contributes to tumor progression through the induction of cancer cell proliferation, chemoresistance, or metastasis^19–22^. We previously explored that haem-dependent dimerization of PGRMC1 accounts for a functional complex that binds to EGF receptor (EGFR) or to cytochromes P450 to regulate cancer proliferation and chemoresistance, respectively^23^. The haem-mediated functional PGRMC1 homodimer is dissociated in response to physiologic levels of carbon monoxide (CO), a gaseous mediator generated by heme oxygenases, and the dissociation of the dimer enhances cancer cell death and chemosensitivity. Several physiological functions of PGRMC1 have been suggested, such as cholesterol biosynthesis^24,25^, amyloid β-induced synaptotoxicity^26,27^, uterine homeostasis^28^, or ovarian follicle development^29^. However, these reports did not unveil the molecular mechanisms by which PGRMC1 regulates cellular and organ phenotypes. Recently, PGRMC1 has been reported to interact with the insulin receptor (IR), and contributes to glucose uptake in cancer cells^30^. Furthermore, it has recently been reported that PGRMC1 interacts with LDL-R and contributes to LDL uptake in cancer cells^31^. These results led us to examine whether PGRMC1 plays a pivotal role in the regulatory mechanisms that trigger adipogenesis.

To prove this hypothesis, we generated adipocyte-specific PGRMC1-knockout mice to examine the roles of PGRMC1 on increased uptake of glucose and lipids. Our study revealed that the PGRMC1 expression is upregulated via the activation of PPARγ or CREB during adipogenesis and provided evidence for crucial roles of PGRMC1 in the interaction between adipogenic stimulators and incorporation of energy substrates to further accelerate adipogenesis and obesity.

## Results

### PGRMC1 is required for lipid accumulation during 3T3L1 cell differentiation

**Fig. 1:**
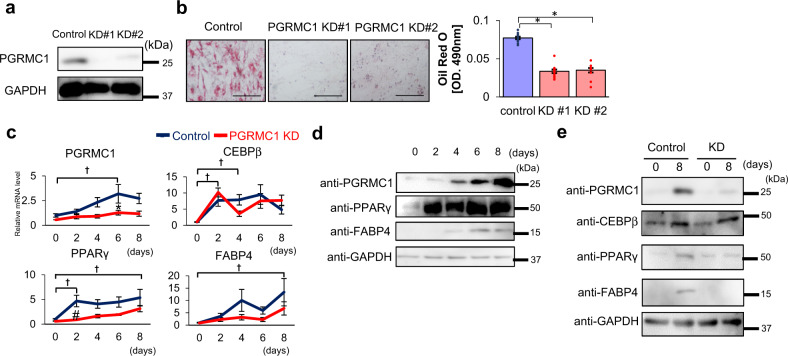
Progesterone receptor membrane-associated component 1 (PGRMC1) is required for lipid accumulation during 3T3L1 cells differentiation. **a** Analyses of protein expressions in differentiated 3T3L1 cells (control, KD#1, or KD#2) by western blotting using antibodies against PGRMC1 or GAPDH. **b** Oil Red O staining of differentiated 3T3L1. Control or two types of stable PGRMC1-KD 3T3L1 cells (PGRMC1 KD#1 and KD#2) were differentiated and stained with Oil Red O. The microscope images are shown in the upper panels of (**b**). Graphs in the lower panel of (**b**) depict the absorbance at 490 nm by Oil Red O (*n* = 8). **c** Analyses of mRNA expression of *PGRMC1, CEBPβ, PPARγ*, or *FABP4* in 3T3L1 cells at the indicated time periods during differentiation (control, PGRMC1 KD) by quantitative PCR (qPCR) (*n* = 10). Analyses of protein expressions in undifferentiated or differentiated 3T3L1 cells (control, KD) by western blotting using antibodies against PGRMC1, CEBPβ, PPARγ, FABP4, or GAPDH. **d** Analyses of protein expressions in 3T3L1 control cells during differentiation by western blotting using antibodies against PGRMC1, PPARγ, FABP4, or GAPDH. **e** Analyses of protein expressions in undifferentiated or differentiated 3T3L1 cells (Control, KD) by western blotting using antibodies against PGRMC1, CEBPβ, PPARγ, FABP4, or GAPDH. Data represent mean ± S.E. Statistical analyses were performed using ANOVA with Tukey’s *T* test. *P < 0.05. ^†^*P* < 0.05 (vs control day 0 (**c**)). ^#^*P* < 0.05 (PGRMC1 KD vs control on the same day (**c**)).

We analyzed the effects of PGRMC1 downregulation on adipose differentiation in a mouse embryonic fibroblast 3T3L1 cell line by using two types (KD#1, KD#2) of stable PGRMC1-knockdown (KD) cells (Fig. 1a). The lipid accumulation detected by Oil Red O staining in differentiated 3T3L1 cells was significantly inhibited by PGRMC1 KD compared to that of control shRNA treated wild-type cells (control) (Fig. 1b). These results suggest that PGRMC1 is required for lipid accumulation during 3T3L1 cell differentiation. We next analyzed the expression levels of PGRMC1 during 3T3L1 cell differentiation by quantitative PCR (qPCR) (Fig. 1c) and western blotting (Fig. 1d). The PPARγ expression was increased at day 2 after induction, and the expression of PGRMC1, similar to FABP4 that is known to be induced by PPARγ, was increased at day 4 after induction, suggesting that PGRMC1 expression, similar to FABP4, is induced after PPARγ activation. When using the PGRMC1-KD cells, the expression of PPARγ and FABP4 was significantly reduced by PGRMC1 KD (Fig. 1e). In contrast with these results, the expression of CCAAT/enhancer-binding protein beta (C/EBP-β), which is known to express at an early stage during adipocyte differentiation^32^, was upregulated at day 2 after induction, and did not change by PGRMC1 KD (Fig. 1e). These results suggested that PGRMC1 expression is upregulated in correlation with activation of PPARγ during adipogenesis. To verify whether PGRMC1 influences adipose differentiation or lipid accumulation, we further analyzed the effect of PGRMC1 KD in 3T3L1 cells stimulated by treatment with activator, thiazolidinedione (TZD). The lipid accumulation in differentiated 3T3L1 cells induced by TZD was significantly inhibited by PGRMC1 KD (Supplementary Fig. 1a). Under these conditions, the induced expressions of PPARγ and FABP4 by TZD did not change by PGRMC1-KD (Supplementary Fig. 1b, c). These indicated that PGRMC1 is required for lipid accumulation during 3T3L1 cell differentiation.

### PGRMC1 expression is enhanced during 3T3L1 cell differentiation

**Fig. 2:**
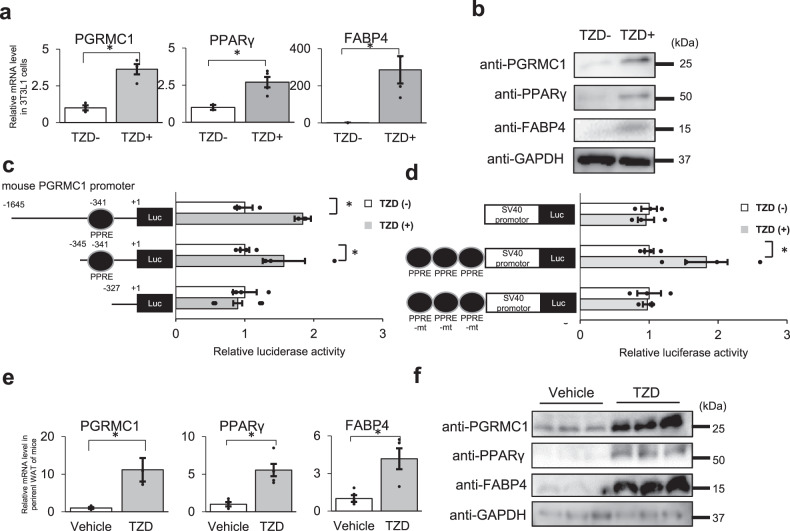
The progesterone receptor membrane-associated component 1 (PGRMC1) expression is enhanced during 3T3L1 cell differentiation. **a** Analyses of mRNA expression in 3T3L1 cells treated with 15 μmol l^−1^ TZD for 2 days by quantitative PCR (qPCR) (*n* = 4). The graph shows relative fold change by normalizing with mRNA levels in 3T3L1 cells treated without TZD. **b** Analyses of protein expressions in 3T3L1 cells treated with 15 μmol l^−1^ TZD by western blotting using antibodies against PGRMC1, PPARγ, FABP4, or GAPDH. **c**, **d** Reporter gene assay of mouse PGRMC1 promoter. The reporter constructs of the *PGRMC1* promoter containing PPRE sequences (−1695/+1-PGRMC1-Luc, -345/+1-PGRMC1-Luc), or lacking PPRE site (-327/+1-PGRMC1-Luc) (**c**), or constructs containing a control SV40 promoter, or three repeats of the PPRE or the mutated PPRE (PPRE-mt) upstream of a control SV40 promoter (**d**) were transfected into 3T3L1 cells and were incubated for 2 days after adding 15 μmol l^−1^ TZD. The graph shows relative luciferase activity by normalizing with luciferase activity in 3T3L1 cells treated without TZD (*n* = 4). **e**, **f** Analyses of the PGRMC1 expression by treatment with TZD in mice. TZD (5 mg kg^−1^) was intraperitoneally injected in C57BL/6J mice for 3 consecutive days. The mRNA expressions of *PGRMC1*, *PPARγ*, and *FABP4* in white adipose tissue (WAT) were analyzed by qPCR (*n* = 5). **e** The protein expressions in perirenal WAT were analyzed by western blotting using antibodies against PGRMC1, PPARγ, FABP4, or GAPDH. **f** Data represent mean ± S.E. Statistical analysis was performed using Student’s *T* test (**a**, **c**, **d**, and **e**). ^*^P < 0.05.

PPARγ acts as a key regulatory transcription factor for the induction of lipid accumulation in adipocytes. We next examined the PGRMC1 expression by treatment with TZD in the 3T3L1 cells. As shown in Fig. 2a, b, the expression of PGRMC1 as well as PPARγ and FABP4 was significantly enhanced by treatment with TZD. These results indicated that the PGRMC1 expression is regulated by PPARγ during adipocyte differentiation. To decipher regulatory mechanisms of *PGRMC1* gene expression, we predicted the transcription factor-binding site in mouse *PGRMC1* promoter sequences using TRANSFAC software. As a result, *PGRMC1* promoter contained the predicted PPARγ response element (PPRE), activating transcription factors (ATF)/cAMP-response element modulator (CREB)-binding element (CRE), glucocorticoid receptor-binding element (GRE), and three SP1-binding elements (Supplementary Fig. 2). Therefore, we performed the reporter gene assay using luciferase constructs containing mouse *PGRMC1* promoter sequences (Fig. 2c). The promoter activities with the PPRE-containing constructs (−1695/+1-PGRMC1-Luc or −345/+1-PGRMC1-Luc) were significantly elevated by treatment with TZD. By contrast, no stimulation was observed after using the construct lacking PPRE site (−327/+1-PGRMC1-Luc). We further confirmed the activity of the predicted PPRE site in the *PGRMC1* promoter by using reporter constructs encoding three repeats of the PPRE (PPRE x3) or the mutated PPRE (PPRE-mt x3) upstream of a control SV40 promoter (Fig. 2d). The reporter activity of the PPRE x3-containing constructs was significantly enhanced by TZD, but no inducible activity was observed when using the control or the PPRE-mutation constructs. We also confirmed that the reporter activities of the PGRMC1 promoter (−1695/+1-PGRMC1-Luc) or the PPRE x3-containing construct were significantly elevated by the addition of TZD in 293T cells co-transfected with the PPARγ expression vector (Supplementary Fig. 3). Furthermore, the *PGRMC1* promoter activity (−1695/+1-PGRMC1-Luc) was enhanced by the addition of insulin (Supplementary Fig. 4a). Insulin signaling is known to induce transactivation of ATF/CREB^33^. The induction of *PGRMC1* promoter activity by insulin was observed when using the CRE-containing construct (−481/+1-PGRMC1-Luc), but not when using the CRE lacking construct (−465/+1-PGRMC1-Luc). Furthermore, we were unable to detect any induction of its promoter activity by dexamethasone (DEX), an inducer of GR (Supplementary Fig. 4b). These results suggested that the *PGRMC1* gene expression was enhanced through mediation by PPARγ and ATF/CREB, which are induced by insulin signaling during adipogenesis. To further examine regulatory mechanisms of *PGRMC1* expression in vivo, TZD was intraperitoneally injected in mice for 3 days, and gene expressions in the WAT were detected by qPCR and western blotting (Fig. 2e, f). The results indicated that the expression level of PGRMC1 in WAT was significantly induced by treatment of TZD.

### PGRMC1 contributed to LDL and VLDL uptake by regulating the translocation of LDL-R and VLDL-R

**Fig. 3:**
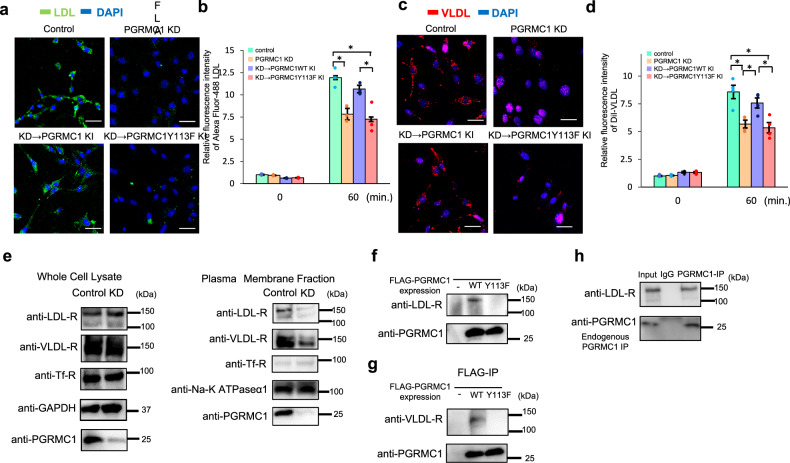
Progesterone receptor membrane-associated component 1 (PGRMC1) contributed to low-density lipoproteins (LDL) and very-low-density lipoproteins (VLDL) uptake by regulating the translocation of LDL-R and VLDL-R. **a**, **b** Analyses of the effect to LDL uptake by PGRMC1. **a** Images of 3T3L1 control cells, PGRMC1 KD cells, or PGRMC1 KD cells expressing shRNA-resistant PGRMC1 WT or PGRMC1-Y113F stained with Alexa Fluor 488-acetylated LDL (green) and DAPI (blue) are shown (scale bar: 10 μm). **b** Flow-cytometric analysis of fluorescence intensities of 3T3L1 control cells, PGRMC1 KD cells, or PGRMC1 KD cells expressing shRNA-resistant PGRMC1 WT or Y113F after incubation with Alexa Fluor 488-acetylated LDL for 60 min. The graph shows the mean of fluorescence intensities (per 10,000 cells) (*n* = 4). **c**, **d** Analyses of the effect to VLDL uptake by PGRMC1. **c** Images of 3T3L1 control cells, PGRMC1 KD cells, or PGRMC1 KD cells expressing shRNA-resistant PGRMC1 WT or PGRMC1-Y113F stained with DiI–VLDL (red) and DAPI (blue) are shown (scale bar: 10 μm). **d** Flow-cytometric analysis of fluorescence intensities of 3T3L1 control cells, PGRMC1 KD cells, or PGRMC1 KD cells expressing shRNA-resistant PGRMC1 WT or Y113F after incubation with DiI–VLDL for 60 min. (*n* = 4). **e** Analyses of regulation of the LDL-R and VLDL-R localization by PGRMC1. Protein expressions in whole-cell lysates were detected by western blotting using antibodies against LDL-R, VLDL-R, Tf-R, GAPDH, or PGRMC1. **f** After plasma membrane fractions of 3T3L1 cells (control and PGRMC1 KD) were extracted, protein expression in the plasma membrane was detected by western blotting using antibodies against LDL-R, VLDL-R, Tf-R, Na-K ATPase α1, or PGRMC1. **f**, **g** Co-immunoprecipitation assay for interaction between PGRMC1 and LDL-R, or PGRMC1 and VLDL-R. FLAG-PGRMC1 WT or Y113F was overexpressed in 3T3L1 cells, and immunoprecipitated with anti-FLAG antibody-conjugated beads. Co-immunoprecipitated proteins were detected with western blotting using anti-PGRMC1, anti-LDL-R antibody, or anti-VLDL-R antibody. **h** Co-immunoprecipitation assay for interaction between endogenous PGRMC1 and LDL-R. 3T3L1 cell lysate was incubated with anti-PGRMC1 antibody or normal rabbit IgG, and then incubated with 10 µl protein A-sepharose beads. Co-immunoprecipitated proteins were detected with western blotting using anti-PGRMC1 or anti-LDL-R antibody. Data are represented as mean ± S.E. Statistical analysis was performed using ANOVA with Tukey’s *T* test (**b**, **d**). ^*^P < 0.05.

Recently, it has been reported that PGRMC1 contributes to LDL uptake through interaction with LDL-R in HeLa cells^31^; however, its regulatory mechanism in adipocyte has remained unclear. Since PGRMC1 was required for lipid accumulation during 3T3L1 cell differentiation (Fig. 1), regulatory roles of PGRMC1 for LDL uptake were further examined in 3T3L1 cells. Control or PGRMC1-KD 3T3L1 cells were treated with Alexa Fluor 488-labeled LDL to analyze the LDL uptake using immunofluorescence microscopy and flow cytometry. Analyses with immunofluorescence microscopy revealed that the Alexa Fluor 488-labeled LDL uptake in PGRMC1-KD cells was significantly suppressed as compared with that in control cells (Fig. 3a and Supplementary Fig. 5a). Furthermore, analyses with flow cytometry showed that the Fluor 488-labeled LDL uptake was significantly decreased in PGRMC1-KD cells than that in control cells (Fig. 3b). We also showed that the LDL uptake in the differentiated 3T3L1 cells induced by TZD was suppressed by PGRMC1 KD (Supplementary Fig. 1d). We further examined the effects of PGRMC1 restoration that was performed by knockdown-rescue procedures using PGRMC1-expressing vectors, in which silent mutations were introduced into the nucleotide sequence targeted by shRNA (Supplementary Fig. 6). As a result, the suppression of LDL uptake by PGRMC1 KD was rescued by expressing shRNA-resistant PGRMC1 WT (Fig. 3a, b). We previously showed that PGRMC1 forms a dimer through heme–heme stacking by 5-coordinate haem with tyrosine 113 residue (Y113) of PGRMC1^23^. To evaluate the effect of the dimerization of PGRMC1 on LDL uptake, we prepared the shRNA-resistant PGRMC1 mutant, Y113F, which was unable to dimerize due to disruption of the haem-binding activity. In contrast to the result using the construct of PGRMC1 WT, the suppression of LDL uptake by PGRMC1 KD was not rescued after using the Y113F mutant, suggesting that haem-dimerized PGRMC1 is required for LDL uptake in 3T3L1 cells. We further analyzed the uptake of the fluorescent Dil-labeled VLDL in control or PGRMC1-KD 3T3L1 cells with immunofluorescence microscopy (Fig. 3c and Supplementary Fig. 5b) and flow cytometry (Fig. 3d). The VLDL uptake was inhibited by PGRMC1 KD, and the suppression was rescued by expressing shRNA-resistant PGRMC1 WT, but not the Y113 mutant. These results collectively suggested that the haem-dimerized PGRMC1 is necessary for facilitating the uptake of LDL and VLDL.

LDL or VLDL is incorporated into the cells via endocytosis mediated by LDL-R or VLDL-R, respectively^34^. To elucidate the mechanism of the regulation of LDL uptake by PGRMC1, we analyzed the cellular distribution of LDL-R or VLDL-R by fractioning plasma membranes of 3T3L1 cells (Fig. 3e). The expression of LDL-R or VLDL-R in the whole-cell lysates did not differ between control and PGRMC1 KD cells (Fig. 3e, left panel). By contrast, the localization of LDL-R or VLDL-R in the plasma membrane was decreased by PGRMC1 KD, whereas it was not changed its localization of plasma membrane protein Na-K ATPase 1, or transferrin receptor (Tf-R), which are known to be regulated by endocytosis^35^. Collectively, these results suggested that the translocation of LDL-R or VLDL-R from the plasma membrane mediated by the haem-dimerized PGRMC1 appears to be responsible for the accumulation of LDL or VLDL in 3T3L1 cells. This finding led us to examine whether dimerized PGRMC1 interacts with LDL-R or VLDL-R in the cells. The FLAG-tagged PGRMC1 expressed in 3T3L1 cells was immunoprecipitated with anti-FLAG antibody-agarose, and the co-immunoprecipitated endogenous LDL-R or VLDL-R was detected. It was observed that the FLAG-tagged PGRMC1 WT interacted with endogenous LDL-R (Fig. 3f) or VLDL-R (Fig. 3g), but not with the Y113F mutant, indicating that the haem-dimerized PGRMC1 interacted with LDL-R and VLDL-R. We also confirmed that LDL-R was co-immunoprecipitated with endogenous PGRMC1 (Fig. 3h). These results suggest that the haem-dimerized PGRMC1 plays a role in the plasma membrane distribution of LDL-R and VLDL-R via direct interaction.

### PGRMC1 contributes to glucose uptake by regulating the translocation of GLUT4 to the plasma membrane

**Fig. 4:**
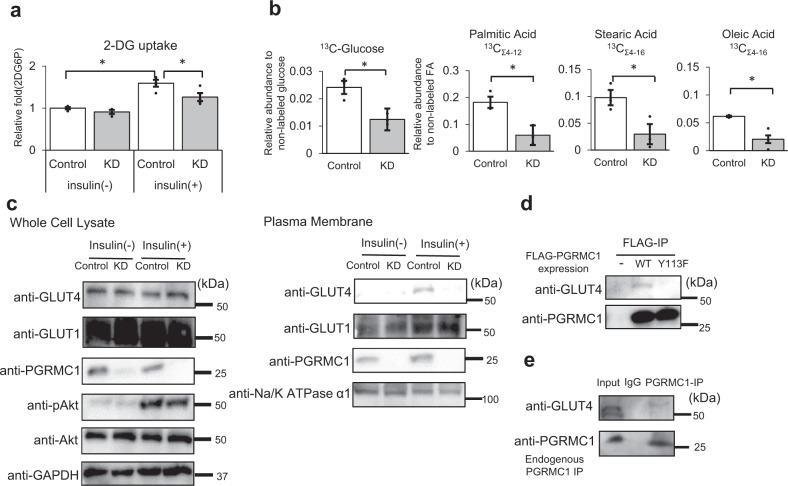
Progesterone receptor membrane-associated component 1 (PGRMC1) contributes to glucose uptake by regulating the translocation to the plasma membrane of GLUT4. **a** Analysis of the effect of insulin-stimulated 2-deoxyglucose (DG) uptake by PGRMC1. After treatment with or without 0.5 μmol l^−1^ insulin for 18 min, 3T3L1 cells (control or PGRMC1 KD) were incubated with 1 μmol l^−1^ 2-DG for 20 min, and the 2-DG uptake was measured. The graph shows relative fold change by normalizing with 2-DG uptake of 3T3L1 control cells without treatment of insulin (*n* = 4). **b** Analysis of the effect of fatty acid synthesis by PGRMC1 using [^13^C_6_]-glucose. After differentiated 3T3L1 cells (control and KD) were incubated with 4.5 g l^−1^ [^13^C_6_]-glucose for 24 h, the fatty acids were extracted. [^13^C_6_]-glucose, [^13^C_4–12_]-palmitic acid, [^13^C_4–16_]-stearic acid, and [^13^C_4–16_]-oleic acid in cells were measured by LC/MS. (*n* = 3). See Supplementary Fig. 7 for more detail. **c** Analyses of regulation of the GLUT4 translocation by PGRMC1. Plasma membrane fractions were extracted from 3T3L1 cells (control and PGRMC1 KD) treated with or without insulin. The expressed proteins in the plasma membrane or whole-cell lysate were detected by western blotting using antibodies against GLUT4, GLUT1, PGRMC1, phosphorylated Akt (pAkt), Akt, Na-K ATPase α1, or GAPDH. **d** Co-immunoprecipitation assay for interaction between PGRMC1 and GLUT4. FLAG-PGRMC1 WT or Y113F was overexpressed in 3T3L1 cells and immunoprecipitated with anti-FLAG antibody-conjugated beads. Co-immunoprecipitated proteins were detected with western blotting using anti-PGRMC1 or anti-GLUT4 antibody. **e** Co-immunoprecipitation assay for interaction between endogenous PGRMC1 and GLUT4. 3T3L1 cell lysate was incubated with anti-PGRMC1 antibody or normal rabbit IgG, and then incubated with 10 µl of protein A-sepharose beads. Co-immunoprecipitated proteins were detected with western blotting using anti-PGRMC1 or anti-GLUT4 antibody. All data are represented as mean ± S.E. Statistical analysis was performed using Student’s *T* test. ^*^P < 0.05.

Glucose uptake in adipocytes plays a crucial role in accumulation of lipids via de novo fatty acid synthesis. Recently, PGRMC1 has been reported to interact with the insulin receptor and to regulate glucose uptake in lung cancer cells^30^. Therefore, we examined the effects of PGRMC1 on glucose uptake by using 2-deoxyglucose (2-DG), an inhibitor of hexokinase, in 3T3L1 cells. The uptake of 2-DG induced by treatment of insulin in 3T3L1 cells was significantly suppressed by PGRMC1 KD, whereas no significant effect by PGRMC1 KD was observed without treatment of insulin (Fig. 4a). We also showed that the 2-DG uptake in the differentiated 3T3L1 cells induced by TZD was suppressed by PGRMC1 KD (Supplementary Fig. 1e). To evaluate the fatty acids synthesis via the incorporation of glucose, 3T3L1 cells were incubated with [^13^C_6_]-glucose for 24 h, and [^13^C_6_]-glucose uptake along with the newly synthesized [^13^C]-labeled fatty acids were analyzed employing LC-MS. As revealed in Fig. 4b and Supplementary Fig. 7, the [^13^C_6_]-glucose, [^13^C]-palmitic, [^13^C]-stearic, and [^13^C]-oleic acid in 3T3L1 cells were significantly decreased by PGRMC1 KD. These results suggested that PGRMC1 contributes to the acceleration of fatty acid synthesis by upregulating glucose uptake in 3T3L1 cells. This observation led us to examine whether PGRMC1 regulates GLUT4 translocation in the plasma membrane. The insulin-induced GLUT4 translocation onto the plasma membrane was inhibited by PGRMC1 KD (Fig. 4c), whereas the GLUT1 translocation was not suppressed by PGRMC1 KD. Furthermore, the insulin-induced Akt phosphorylation was not changed by PGRMC1 KD, suggesting that suppression of the GLUT4 translocation by PGRMC1 KD is independent of the Akt signaling induced by insulin. We thus examined whether PGRMC1 binds to GLUT4 using a co-immunoprecipitation assay. The FLAG-tagged PGRMC1 expressed protein in 3T3L1 cells was immunoprecipitated, and the co-immunoprecipitated endogenous GLUT4 was detected (Fig. 4d). WT-PGRMC1, but not Y113F mutant, which lacks the ability to bind heme for dimerization, interacted with endogenous GLUT4. We also confirmed that GLUT4 was co-immunoprecipitated with endogenous PGRMC1 (Fig. 4e). These results suggested that the dimerized PGRMC1 was directly bound to GLUT4, thereby regulating its translocation to the plasma membrane, which in turn facilitated glucose uptake, resultant de novo fatty acid synthesis in 3T3L1 cells.

### CO interferes with the PGRMC1-mediated lipid and glucose uptake in adipocyte

**Fig. 5:**
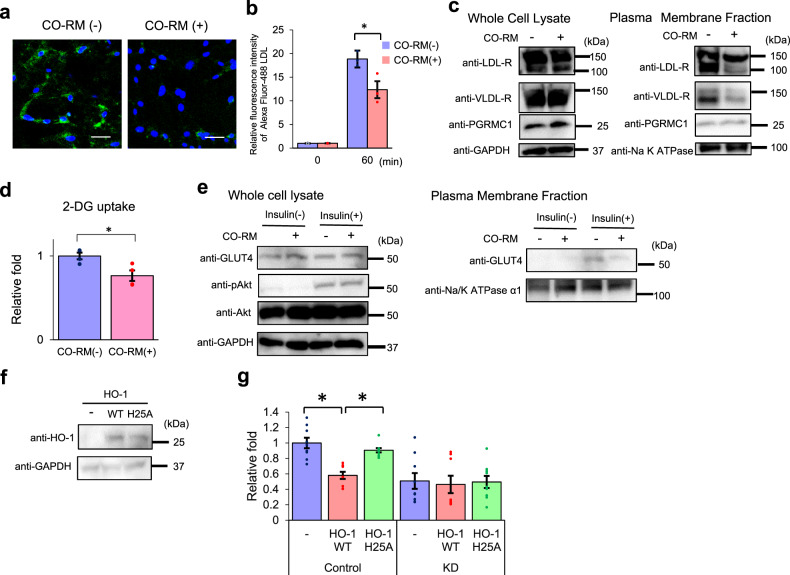
Carbon monoxide (CO) interferes with low-density lipoproteins (LDL) and glucose uptake. **a**, **b** Analyses of the effect to LDL uptake by CO-RM. 3T3L1 cells were incubated with 10 μmol l^−1^ CO-RM or control RuCl_3_ for 2 h, then treated with 5 mg l^−1^ Alexa Fluor 488-acetylated LDL. **a** Images of 3T3L1 cells treated with or without CO-RM and stained with Alexa Fluor 488-acetylated LDL (green) and DAPI (blue) are shown (scale bar: 10 μm). **b** Flow-cytometry analysis of the fluorescence intensities of 3T3L1 cells after incubation with Alexa Fluor 488-acetylated LDL for 60 min. The graph shows the mean of fluorescence intensities (per 10,000 cells) (*n* = 4–5). **c** Analyses of regulation of the LDL-R or VLDL-R localization by CO-RM. Plasma membrane fraction or whole-cell lysate of 3T3L1 cells with or without CO-RM were analyzed by western blotting using antibodies against LDL-R, VLDL-R, or GAPDH. **d** Analysis of the effect of 2-DG uptake by CO-RM. After treatment of 0.5 μmol l^−1^ insulin for 18 min with or without 10 μmol l^−1^ CO-RM for 2 h, 3T3L1 cells were incubated with 1 μmol l^−1^ 2-DG for 20 min, and the 2-DG uptake was measured. The graph shows relative fold change by normalizing with 2-DG uptake of cells without CO-RM (*n* = 4). **e** Analyses of regulation of the GLUT4 translocation by CO-RM. Plasma membrane fraction of 3T3L1 cells (with or without treatment with CO-RM) was incubated with or without 0.5 µmol l^−1^ insulin for 2 h. Protein expressions in the plasma membrane or whole-cell lysate were detected by western blotting using antibodies against either GLUT4, progesterone receptor membrane-associated component 1 (PGRMC1), pAkt, Akt, Na-K ATPase α1, or GAPDH. **f** 3T3L1 cells were transiently transfected with the shRNA-resistant expression vector of wild-type haem oxygenase 1 (HO-1) (WT) or the H25A mutant (H25A). The protein expressions were analyzed by western blotting using antibodies against HO-1 or GAPDH. **g** Analysis of the effect of HO-1 to 2-DG uptake. 2-DG uptake was measured in 3T3L1 cells (control or PGRMC1 KD) expressing HO-1 WT or H25A mutant. All data represent as mean ± S.E. Statistical analysis was performed using ANOVA with Tukey’s *T* test. ^*^P < 0.05.

Haem-dimerized PGRMC1 interacted with LDL-R and GLUT4 and contributed to cellular uptake of LDL/VLDL and glucose (Figs. 3 and 4). We have previously shown that physiological actions of carbon monoxide (CO) derived from haem oxygenase (HO)^36–39^, and PGRMC1 accounts for a receptor of CO which interferes with the haem-stacking dimerization of PGRMC1^23^, raising a possibility that CO interferes with the PGRMC1-mediated lipid and glucose uptake in the adipocyte. To examine whether CO interferes with LDL uptake, we examined the effect of LDL uptake in 3T3L1 cells by treating the cells with a CO-releasing molecule (CO-RM)^37,40^. Analyses with fluorescence microscopy or flow cytometry revealed that the fluorescent LDL uptake was decreased by the addition of the CO-RM (Fig. 5a, b). Furthermore, the plasma membrane distribution of LDL-R and VLDL-R was inhibited by treatment with CO-RM (Fig. 5c). We also showed that treatment with CO-RM inhibited the 2-DG uptake (Fig. 5d). The PGRMC1-mediated GLUT4 translocation in the plasma membrane was inhibited by treatment of CO-RM, but not in the whole-cell lysates (Fig. 5e). To evaluate the role of CO gas, regulation of glucose uptake in 3T3L1 cells was analyzed by ectopically expressing haem oxygenase 1 (HO-1), and its H25A mutant lacking the catalytic activity^39^ (Fig. 5f). As shown in Fig. 5g, the uptake of 2-DG in 3T3L1 control cells was significantly suppressed by expression of HO-1 WT, but not by that of the H25A mutant. The inhibitory effect of 2-DG uptake by HO-1 expression was at the same level as that by PGRMC1 KD. By contrast, no inhibitory effect by HO-1 expression was observed in the PGRMC1 KD 3T3L1 cells. In addition, the fluorescent LDL uptake or the 2-DG uptake in 3T3L1 cells was significantly elevated by the addition of haemin and the elevation of the LDL or 2-DG uptake by haemin was not observed in the PGRMC1 KD cells (Supplementary Fig. 8a, b). These results suggested that CO interferes with the uptake of lipid and glucose uptake in adipocyte, by interfering with the heme-mediated PGRMC1 dimerization. A recent report showed that PGRMC1 in mitochondria contributes to regulating haem biosynthesis via interaction with ferrochelatase, which catalyzes the insertion of ferrous iron into protoporphyrin IX^41^. Furthermore, it has been reported that PGRMC1 in mitochondria-associated membranes transfers haem to PGRMC2, which is highly homologous to PGRMC1 and is required for thermogenesis in brown adipocyte^42^. Therefore, we analyzed the cellular distribution of PGRMC1 in 3T3L1 cells (Supplementary Fig. 9a, b). PGRMC1 mainly localized in the perinuclear region in 3T3L1 cells, and its distribution was observed in both of ER (Concanavalin A staining) and mitochondria (MitoTracker). No significant change of PGRMC1 distribution was observed by the addition of insulin, haemin, or CO-RM. This suggests that PGRMC1 located in the ER contributes to nutrients uptake in adipocyte by regulating protein transport of low-density lipoprotein receptors (LDL-R) and GLUT4 to the plasma membrane, independently of its function of haem biosynthesis in the mitochondria.

### PGRMC1 contributes to the progression of adipocyte hypertrophy in mice

**Fig. 6:**
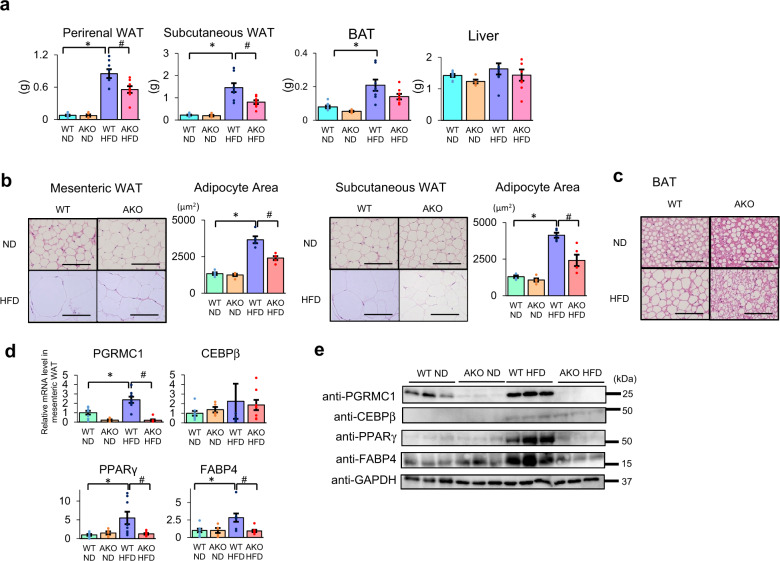
Progesterone receptor membrane-associated component 1 (PGRMC1) contributes to the progression of adipocyte hypertrophy in mice. **a** Tissue weights of perirenal white adipose tissue (WAT), subcutaneous WAT, brown adipose tissue (BAT), and liver in C57BL/6J mice (WT or PGRMC1 adipose tissue-specific knockout (AKO)) fed normal diet (ND) or high-fat diet (HFD) for 16 weeks. (*n* = 5–8). **b** Paraffin sections of mesenteric WAT and subcutaneous WAT from WT and PGRMC1-AKO mice fed ND or HFD stained with hematoxylin and eosin (scale bar: 500 μm). The graph depicts the average adipocyte area (at least 100 cells per mouse) (*n* = 5). **c** The paraffin sections of BAT in WT and PGRMC1-AKO mice fed ND and HFD stained with hematoxylin and eosin (scale bar: 500 μm). **d** Analyses of mRNA expressions in mesenteric WAT of WT and PGRMC1-AKO mice fed ND or HFD by quantitative PCR (qPCR) (*n* = 5–8). The graph shows relative fold change after normalizing with the mRNA level of *GAPDH*. **e** The analyses of protein expressions in mesenteric WAT of WT and PGRMC1-AKO mice fed ND or HFD by western blotting using the antibody against PGRMC1, CEBPβ, PPARγ, FABP4, or GAPDH. Data are represented as mean ± S.E. Statistical analysis was performed using ANOVA with Tukey’s *T* test. ^*^P < 0.05; ^**^P < 0.01; ^***^P < 0.001.

To determine roles of PGRMC1 in adipose tissue in vivo, we generated PGRMC1 *flox/flox* mice in which PGRMC1 exon 2 was flanked with two flox sites in C57BL/6J (WT) mice, and crossed them with Adiponectin-Cre mice^43^ to create adipose tissue-specific PGRMC1-knockout (AKO) mice (Supplementary Fig. 10a). We confirmed that the PGRMC1 expression was specifically disrupted in WAT of the PGRMC1-AKO mice by western blotting (Supplementary Fig. 10b). Next, we examined the adipose tissue progression of WT or PGRMC1-AKO mice fed with normal diet (ND) or high-fat diet (HFD) for 12 weeks. Under these conditions, the amounts of food intake did not change between WT and PGRMC1-AKO mice fed HFD (Supplementary Fig. 11a). As shown in Fig. 6a, while the weights of WAT (perirenal and subcutaneous) and brown adipose tissue (BAT) in WT mice fed HFD significantly increased compared to those fed ND, such increases were suppressed in PGRMC1-AKO mice. In addition, the liver weights did not change between WT and AKO mice fed either ND or HFD. The body weights of PGRMC1-AKO mice were significantly decreased compared to those of WT mice irrespective of being fed with either ND or HFD (Supplementary Fig. 11b), suggesting that PGRMC1-KO mice exhibit a reduction of body weight gain, at least in part due to reduced adipose tissue development. The plasma membrane translocation of LDL-R and GLUT4 in WAT with HFD was decreased by PGRMC1 AKO (Supplementary Fig. 12). Analyses of blood biochemistry revealed that the levels of LDL/VLDL cholesterol and TG induced by HFD were elevated in PGRMC1-AKO mice compared to WT mice (Table 1). This suggested that suppression lipid uptake such as LDL and VLDL by disruption of PGRMC1 in adipocytes resulted in an increase in blood lipids. We also conducted glucose tolerance test (GTT); however, there was no significant difference between WT mice and PGRMC1-AKO mice (Supplementary Fig. 13). Serum insulin levels were elevated in mice fed HFD compared to mice fed ND, but did not change between WT and AKO mice fed either ND or HFD (Table 1). Analyses using a metabolic cage showed that no significant change of V_O2_, respiratory quotient (RQ), heat production, and voluntary activity was observed between WT and PGRMC1-AKO mice during light or dark cycles (Supplementary Fig. 14).

**Table 1:** Analyses of blood biochemistry of WT and PGRMC1-AKO mice fed ND or HFD.

	WT ND	AKO ND	WT HFD	AKO HFD
TG	54.4 ± 5.6	47.1 ± 2.9	50.2 ± 4.1	81.3 ± 7.1^#,†^
Total cholesterol	109.7 ± 1.1	110.0 ± 1.6	134.2 ± 1.3^#^	132.1 ± 3.7^#^
HDL cholesterol	99.5 ± 1.8	96.7 ± 1.3	109.1 ± 1.2	99.3 ± 1.1
LDL/VLDL cholesterol	10.2 ± 2.0	13.3 ± 2.3	25.1 ± 0.9^#^	32.8 ± 2.5^#,†^
Glucose (12 weeks)	160.8 ± 4.3	163.6 ± 10.3	189.8 ± 20.3	194.3 ± 10.6
Glucose (16 weeks)	216.8 ± 9.5	221.0 ± 11.2	262.8 ± 7.2^#^	277.4 ± 14.2^#^
Insulin	1.1 ± 0.3	1.9 ± 0.8	8.6 ± 2.0^#^	8.4 ± 2.5^#^
AST	18.9 ± 1.5	25.6 ± 0.8	40.1 ± 5.6^#^	38.7 ± 3.9
ALT	11.4 ± 0.8	15.2 ± 0.6	24.1 ± 6.5	22.8 ± 5.5

*LDL* low-density lipoproteins, *VLDL*, very-low-density lipoproteins, *HDL* high-density lipoproteins, *PGRMC1* progesterone receptor membrane-associated component 1, *ND* normal diet, *HFD* high-fat diet, *TG* triglycerides.

Plasma biochemical parameters were analyzed using WT and PGRMC1-AKO mice after 16 weeks fed ND or HFD. Data are represented as mean ± S.E. Statistical analysis was performed using ANOVA with Tukey’s *T* test. ^#^*P* < 0.05 (mice fed HFD vs mice fed ND). ^†^*P* < 0.05 (AKO mice fed HFD vs WT mice fed HFD).

We subsequently analyzed adipose tissue histologically. As observed in Fig. 6b, adipocyte hypertrophy was observed in mesenteric and subcutaneous WAT of WT mice fed HFD; however, the hypertrophy was significantly suppressed in PGRMC1-AKO mice fed HFD. In BAT tissues, the formation of large lipid droplets was observed in WT mice fed HFD, but it was suppressed in PGRMC1-AKO mice (Fig. 6c). In the liver tissue, significant lipid accumulation was observed in both the WT and PGRMC1-AKO mice fed HFD (Supplementary Fig. 15a). Supporting this, intrahepatic contents of TG in PGRMC1-AKO mice fed HFD were unchanged as compared with those in the WT mice (Supplementary Fig. 15b). Further analyses of the adipose tissues were carried out to compare alterations in the expression of genes related to lipid metabolism between WT and PGRMC1-KO mice. In WAT, expression of *PGRMC1* was significantly enhanced in WT mice by HFD, which was similar as compared to that in WT mice treated with TZD (Fig. 6d, e). In addition, expression of *PPARγ* or *FABP4* was also enhanced significantly in WT mice by HFD, but not in PGRMC1-AKO mice (Fig. 6d, e). This suggested that inhibition of *PGRMC1* downregulated the *PPARγ* or *FABP4* expression through the reduction of lipid accumulation in WAT, while *PPARγ* was an upstream factor of *PGRMC1*. In BAT, we analyzed not only the expression of *PGRMC1* and *PPARγ* but also the expression of genes responsible for thermogenesis (*PPARα*, *PGC1-α*, *UCP1*, and *CPT1b*) (Supplementary Fig. 16a). In contrast to the results in WAT, the *PGRMC1* expression in BAT was unchanged in WT mice fed HFD, and the expressions of *PPARγ*, *PPARα*, *PGC1-α*, and *Cpt1b* were not altered in PGRMC1-AKO. The gene expression levels of *PGRMC1*, *PPARγ*, and *LDL-R* in the liver did not change between WT and PGRMC1 AKO (Supplementary Fig. 16b). Thus, these data suggest that HFD enhances the PGRMC1 expression in WAT and contributes toward the progression of WATs to cause obesity.

## Discussion

PGRMC1 is highly expressed in a variety of cancer cells and is involved in their proliferation and chemoresistance; however, its physiologic roles or regulation of PGRMC1 expression have remained largely unknown. This study revealed that the PGRMC1 is induced in adipocytes to regulate adipogenesis through mechanisms involving transcription factors such as PPARγ and CREB/ATF via PPRE or CRE of the regulatory regions of the *PGRMC1* gene. In mice, the expression of PGRMC1 in WAT is enhanced by HFD or by treating with TZD. However, its expression in the liver and BAT is not altered by HFD, suggesting that the action of PGRMC1 in the regulation of adipogenesis occurs specifically in WAT. The PPARγ expression was also enhanced in WAT by HFD but not in the liver and BAT, suggesting that induction of the PPARγ expression enhances the PGRMC1 expression in vivo. On the other hand, the PPARγ expression was reduced by PGRMC1 KD in 3T3L1 cells or by PGRMC1 KO in adipose tissues. It has been reported that the PPARγ expression in 3T3-F442A cells was downregulated by knockdown of CD36, which is required for lipid accumulation by upregulating fatty acid uptake^44^. This suggests that inhibition of PGRMC1 downregulates the PPARγ expression by reducing lipid accumulation in adipocytes. PPARγ is known to regulate the lipid accumulation by transactivating the expression of several genes, such as for LDL-R^45^, lipoprotein lipase (LPL), and CD36^46^ for facilitating the uptake of lipids, or GLUT4^7,8^ for stimulating glucose uptake. However, the intervening mechanisms by which PPARγ simultaneously stimulates both LDL-R-mediated lipid uptake and the GLUT4-dependent fatty acid synthesis remain to be unveiled. This study revealed that the expression of PGRMC1 is induced by insulin and/or PPARγ to stimulate lipid accumulation in adipocytes. In addition, the C/EBP-β expression, early-stage marker during adipocyte differentiation, was not affected by suppression of PGRMC1. Therefore, our results indicate that *PGRMC1* is a novel target gene regulated by insulin and PPARγ for lipid accumulation in the adipocyte.

To date, PGRMC1 is known to interact with several proteins, including EGFR^21,23^, cytochromes P450^25,47^, plasminogen activator inhibitor RNA-binding protein 1 (PAIR-BP1)^48^, or tubulin^49^. We previously reported that the haem-mediated dimer of PGRMC1 is necessary for the interaction with these molecules, such as EGFR and cytochrome P450s^23^. This study showed that haem-dimerized PGRMC1 interacts with LDL-R or VLDL-R and regulates their localization on the plasma membrane. It is known that LDL-R or VLDL-R translocates from the ER or endosome to the plasma membrane, and regulates LDL or VLDL uptake via endocytosis^11–13^. It has been shown that PGRMC1, which is located in the ER and endosomes, is thought to play an important role in regulating the intracellular protein translocation, as PGRMC1 contains several YXXϕ motifs that are implicated in vesicle transport and endocytosis^50–52^. Therefore, our results suggested that PGRMC1 would contribute to the plasma membrane translocation of interacting proteins such as LDL-R or VLDL-R via the regulation of vesicle transport. Recently, Bartuzi et al.^53^ showed that the COMMD/CCDC22/CCDC93 (CCC) and the Wiskott–Aldrich syndrome protein and SCAR homolog (WASH) complexes regulate endosomal sorting and lysosomal degradation of LDL-R. Although further study is needed, PGRMC1 might involve in such regulation of endosomal trafficking of LDL-R or VLDL-R.

We showed that haem-dimerized PGRMC1 also interacts with GLUT4, and regulated its translocation into the plasma membrane to facilitate glucose uptake in adipocytes. Recently, it has been reported that PGRMC1 directly interacts with insulin receptor (IR) in lung cancer cells, but PGRMC1 knockdown did not change the phosphorylation of Akt, which is downstream of IR^30^. Supporting this report, our analysis also revealed that PGRMC1 did not affect the Akt phosphorylation induced by insulin in 3T3L1 cells. These results suggested that the direct interaction of PGRMC1 with GLUT4 contributed to the regulation of GLUT4 translocation, leading to stimulation of glucose uptake in adipocytes. In addition, PGRMC1 knockdown did not affect plasma membrane localization of GLUT1, which is constitutive glucose transporter. These suggest that PGRMC1 could regulate insulin-induced glucose uptake by promoting GLUT4 translocation in the adipocyte.

Another important result in this study is that either HO-1 induction or CO interferes with the uptake of LDL and glucose in 3T3L1 cells. There have been considerable numbers of experimental data showing that obesity is suppressed by the induction of HO or by the administration of CO-RM^54–57^. However, reception mechanisms of CO and mechanisms by which CO suppresses obesity remained largely unknown. While further studies are necessary to inquire which endogenous HO product, CO or biliverdin/bilirubin, is responsible for attenuation of obesity in vivo^58^, the current observation showing crucial roles of heme-mediated PGRMC1 dimerization led us to suggest that CO serves as a modulator of adipogenesis to inhibit plasma membrane translocation of LDL/VLDL-R and GLUT4 by dissociating the heme-mediated dimer. Therefore, investigation to unveil the HO-1/CO/PGRMC1 axis deserves further translational studies to control obesity and adipocyte hypertrophy.

In a mice study, we demonstrated that PGRMC1-AKO mice reduced the increases of fat mass and adipocyte hypertrophy induced by HFD, suggesting that PGRMC1 deficiency decreases the lipid storage in adipose tissue. PGRMC1-AKO did not affect the TG and LDL/VLDL cholesterol in the serum of the mice fed ND, but their levels were increased in the mice fed HFD. Whereas PGRMC1 contributed to GLUT4-regulated glucose uptake in 3T3L1 cells, no significant difference was observed between WT mice and PGRMC1-AKO mice by GTT analysis. Given that the skeletal muscle is known to be the major tissue for insulin-regulated glucose uptake (80–95%)^59,60^, the regulation of glucose uptake in adipocytes by PGRMC1 would not result in significant changes of blood glucose. Collectively, these results suggest that PGRMC1 is enhanced by HFD, leading to promoting the HFD-induced lipid accumulation in adipose tissue. PGRMC2 has been recently reported to contribute to brown adipocyte progression and thermogenesis^42^. PGRMC1 was suggested to transfer haem to PGRMC2 in mitochondria-associated membranes in order to upregulate its function. The adipocyte-specific PGRMC2 knockout mice showed decreased BAT mass, thermogenesis, or expression of thermogenic genes, such as UCP1 or PGC1α. However, such HFD-fed mice showed no significant change in body weight or WAT mass^42^. We observed during our analyses that PGRMC1 AKO significantly decreased the body weight or WAT and BAT mass without any significant suppression of thermogenesis and UCP1 or PGC1α expressions in the BAT. Although further PGRMC1 functional analyses would be required in the BAT, these results indicate that PGRMC1 contributes to fat accumulation in the WAT through a PGRMC2-independent mechanism.

**Fig. 7:**
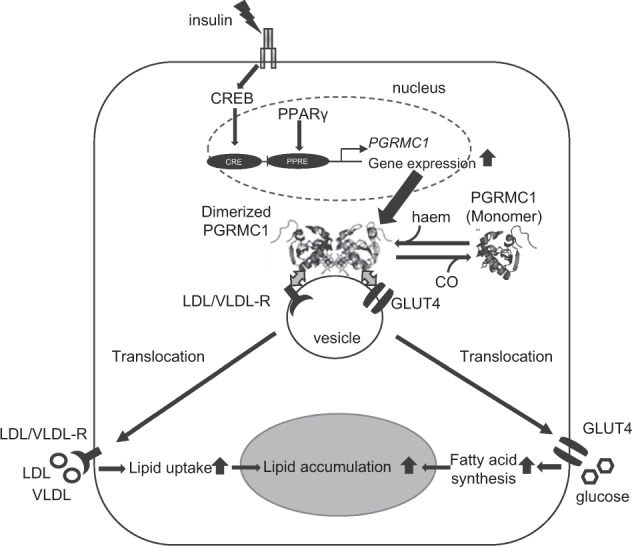
Schematic model for the regulation of lipid accumulation in adipocytes by progesterone receptor membrane-associated component 1 (PGRMC1). When insulin signaling induces adipogenesis, the *PGRMC1* gene expression is transactivated by ATF/CREB and PPARγ. The heme-dimerized PGRMC1 interacts with LDL-R, VLDL-R, or GLUT4 and facilitates their translocation to the plasma membrane. Consequently, PGRMC1 contributes to lipid accumulation in adipocytes by regulating the lipid uptake via LDL-R or VLDL-R, or de novo fatty acid synthesis.

In conclusion, this study presents the pivotal roles of heme-mediated PGRMC1 dimer formation in regulating lipid accumulation in adipocytes. The model for the regulatory mechanisms by PGRMC1 is illustrated in Fig. 7. When insulin signaling induces adipogenesis, the *PGRMC1* gene expression is transactivated by CREB and PPARγ. The heme-dimerized PGRMC1 interacts with LDL-R or GLUT4, and facilitates their translocation to the plasma membrane, followed by the facilitation of lipid uptake via LDL-R or by de novo fatty acid synthesis in the adipocytes. Thus, PGRMC1 contributes to the development of obesity via lipid accumulation in adipocytes. In addition, our results using PGRMC1-AKO mice suggest that PGRMC1 regulates lipid accumulation, specifically in the adipocytes, without affecting the phenotype of the whole body. This study thus sheds light on the possibility of developing therapeutic interventions against obesity and its associated metabolic dysfunction by targeting the PGRMC1 function in adipocytes.

## Methods

### Materials

Dulbecco’s modified Eagle’s medium (DMEM), insulin, dexamethasone, troglitazone, protease inhibitor cocktail III, and diamidinophenylindole (DAPI) were purchased from Wako, Osaka, Japan. Bovine serum (BS) and Alexa fluor 488-acetylated LDL were purchased from Life Technologies, Carlsbad, CA. DiI–VLDL was purchased from Kalen Biomedical, Germantown, MD. Blasticidin and fetal bovine serum (FBS) were purchased from Biowest, Nuaille, France. Isobutylmethylxanthine (IBMX), CO-releasing molecule 3 (CO-RM3), FLAG peptide, anti-FLAG antibody-conjugated agarose and [^13^C_6_]-glucose were purchased from Sigma-Aldrich, St. Louis. 2-deoxyglucose (2-DG) was purchased from the Tokyo chemical industry, Tokyo, Japan.

### Antibodies

The antibodies were purchased from the following manufacturers: anti-GAPDH antibody (Santa Cruz Biotechnology: sc-25778), anti-PGRMC1 antibody for western blotting (Cell Signaling: 13856S), anti-PGRMC1 antibody for immunofluorescent staining (Abcam: ab48012), anti-PPARγ antibody (Abcam: ab59256), anti-FABP4 antibody (Abcam: ab66682), anti-LDL-R antibody (R&D: AF2255), anti-VLDL-R antibody (R&D: AF2258), anti-Tf-R antibody (Abcam: ab84039), anti-Na-K ATPase α1 antibody (Abcam: ab7671), anti-GLUT1 antibody (Abcam: ab115730), anti-GLUT4 antibody (Abcam: ab33780), anti-Akt antibody (Cell Signaling: #9272S), anti-pAkt antibody (Cell Signaling: #4060S), and anti-HO-1 antibody (Santa Cruz Biotechnology: sc-136960).

### Constructions of plasmids

Mouse PGRMC1 cDNA was cloned from cDNA library of 3T3L1 cells using the primers (forward: 5′-TTTGAATTCATGGCTGCCGAGGATGTGGTG-3′, reverse: 5′-TTTGGATCCTTCATTCTTCCGAGCTGTCTC-3′), digested with Eco RI and BamHI, and then ligated into the C-terminus FLAG-epitope-tagged expression vector, p3xFLAG-CMV-14 (Sigma). The Y113F mutant of mouse PGRMC1 was prepared using the primers (sense: 5′-CCTGAGGGGCCATTTGGGGTCTTTGGC-3′, antisense: 5′-GCCAAAGACCCCAAATGGCCCCTCAGG-3′). The PGRMC1 fragment containing resistant sequences for shRNA (KD#1) was prepared using the primers (sense: 5′-CTCTGCATCTTTCTACTATATAAGATCG-3′, antisense: 5′-CGATCTTATATAGTAGAAAGATGCAGAG-3′).

Mouse PGRMC1 promoter gene (−1645~+1) was cloned from the mouse genomic library (Clontech) using primers (forward: 5′-TTTGCTAGCATGTGGTTGCTGGGATTTGAAC-3′, reverse: 5′-TTTAAGCTTGATCTCTGGAGCAAAGGTTGG-3′), digested with Nhe I and HindIII, and subsequently ligated into the luciferase reporter vector pGL3-Basic (PROMEGA). The truncated sequences of mouse PGRMC1 promoter were prepared using primers (5′-TTTGCTAGCCATTTTAACTGACGTCATTTG-3′ for -481, 5′-TTTGCTAGCGCCGCGAGGAGCGGGCCCTAA-3′ for -466, 5′-TTTGCTAGCAACCGGGGCAGAGGCCCAGCA-3′ for -345, and 5′-TTTGCTAGCAGGTAGAACAGAGAGGGGGCT-3′ for -327). For the construction of the PPRE-containing reporter plasmids, three tandem-repeated PPRE fragments (5′-ACCGGGGCAGAGGCCCAGC-3′ × 3) or mutated fragments (5′-ACCGGGTTTGAGGGTTTAGC-3′ × 3) were introduced into the Nhe I/Xho I site upstream of the SV40 promoter region of pGL3-control vector (PROMEGA).

Human HO-1 (wild-type and H25A mutant) cDNA was amplified with the primers (hHO-1 Fwd-HindIII: 5′-CCCCAAGCTTATGCCTTCTGAGACCCCC-3′, hHO-1 Rev-BamHI: 5′-CGGGATCCTCACATGGCATAAAGCCC-3′). PCR fragments were digested with HindIII/BamHI, and then ligated into p3xFLAG-CMV10 vector (SIGMA).

### Cell culture and treatments

3T3L1 cells were maintained in DMEM containing 10% bovine serum (BS) at 37 °C under 5% CO_2_. To prepare two types of stable PGRMC1-knockdown (PGRMC1 KD) cell lines, 3T3L1 cells were transfected with lentiviruses encoding an shRNA sequence targeting PGRMC1, according to the manufacturer’s instructions (Life Technologies, Carlsbad, CA). Stable cell lines were selected by maintaining the cells in a medium supplemented with 10 mg l^−1^ blasticidin for 1 week.

Differentiation of 3T3L1 cells was induced by changing the culture medium to DMEM containing 10% FBS, 0.25 µmol l^−1^ dexamethasone, 0.5 mmol l^−1^ IBMX, and 0.2 µmol l^−1^ insulin (day 0). After 2 days, the culture medium was changed to DMEM containing 10% FBS and 0.2 µmol l^−1^ insulin (day 2). Two days later (day 4), the medium was changed to DMEM containing 10% FBS. Thereafter, the medium was changed to DMEM containing 10% FBS every day. The differentiated 3T3L1 cells were analyzed on day 8 after inducing differentiation.

To determine the effect of thiazolidinedione (TZD), troglitazone dissolved in ethanol was added to the culture medium for the TZD group at a final concentration of 15 µmol l^−1^, and the cells were incubated for 48 h. For the analysis of the effect by TZD on 3T3L1 differentiation, 3T3L1 cells were treated with 15 µmol l^−1^ TZD every 2 days in addition to differentiation inducers as described above.

For the analysis of LDL uptake, cells were incubated in serum-free DMEM supplemented with Alexa Fluor 488-acetylated LDL to a final concentration of 5 mg l^−1^ at 37 °C for 60 min. For the analysis of VLDL uptake, cells were incubated in serum-free DMEM supplemented with DiI–VLDL to a final concentration of 10 mg l^−1^ at 37 °C for 60 min.

For the analysis of expression in plasma expression, plasma membrane proteins were prepared using a plasma membrane isolation kit (Invent Biotechnologies, Inc., Plymouth, MN, USA). Briefly, 3T3L1 cells were collected and lysed in buffer A. The lysate was centrifuged at 16,000 × *g* for 10 min, and the pellet was re-suspended in buffer B and centrifuged at 7,800 × *g* for 5 min. The supernatant was further centrifuged at 16,000 × *g* for 15 min, and the pellet as plasma membrane protein fraction was used for western blotting.

For the analysis of the effect of CO, CO-RM3 or ruthenium chloride (RuCl_3_) was added to a culture medium at a final concentration of 10 μmol l^−1^, and the cells were incubated for 2 h.

For the analysis of 2-DG uptake, cells were incubated with serum-free medium overnight. After treatment with or without 0.5 μmol l^−1^ insulin for 18 min, the cells were incubated with 1 µmol l^−1^ 2-DG for 20 min. 2-DG uptake was analyzed by using a 2-DG Uptake Measurement Kit (Cosmo Bio Co., Ltd., Tokyo, Japan), according to the manufacturer’s instructions. For the analysis of GLUT4 translocation and insulin signaling, the cells were incubated in a serum-free medium overnight and later incubated with or without 0.5 µmol l^−1^ insulin for 5 min (insulin signaling) or for 2 h (GLUT4 translocation).

For the analysis of the effect of haem oxygenase, 3T3L1 cells were transfected with human HO-1 vector (p3xFLAG-CMV10, Sigma, Tokyo, Japan) using lipofectamine 2000 (Life Technologies, Carlsbad, CA). For the analysis of the effect of haemin, hemin was added to a culture medium at a final concentration of 10 μmol l^−1^ for 1 h.

### Oil Red O staining

Differentiated 3T3L1 cells were washed with phosphate buffered saline (PBS) and then fixed with 10% neutral formaldehyde for 2 h. The fixed cells were stained with Oil Red O (5 g l^−1^ Oil Red O in 99% isopropanol:water, 6:4) for 40 min, and subsequently observed with a microscope (ECLIPSE E600, Nikon, Tokyo, Japan). Oil Red O was extracted with 99% isopropanol for 5 min, and absorbance of the isopropanol solution at 490 nm was measured with a spectrophotometer (Benchmark Plus, Bio-Rad, Richmond, CA).

### Analyses of qPCR for mRNA expression

The total RNA from 3T3L1 cells or mice tissue was extracted using a TRIzol reagent kit (Life Technologies, Carlsbad, CA) according to the manufacturer’s instructions. Reverse transcription was performed using a PrimerScript RT reagent kit (Takara Bio Inc., Shiga, Japan). The mRNA was quantified by performing using a specific primer and TB Green Fast qPCR Mix (Takara Bio Inc., Shiga, Japan) with real-time PCR System (Applied Biosystems^TM^7000, Life Technologies, Carlsbad, CA). The relative mRNA level was standardized with GAPDH. Analyses of the samples were carried out in duplicate by qPCR. The sequences of primers used are in Supplementary Table 1.

### Luciferase reporter assays

3T3L1 cells were transfected with reporter constructs of the PGRMC1 promoter and Renilla-luciferase vector (pRL-CMV, Promega, Madison, WI) using lipofectamine 2000 (Life Technologies, Carlsbad, CA). The transfected cells were incubated in DMEM containing 10% FBS for 24 h, then in DMEM containing 10% FBS with or without treatment with 15 µmol l^−1^ TZD for 48 h. Luciferase activities were measured with a luminometer (Cytation 5, BioTek Instruments, Inc., Winooski, VT) using a dual-luciferase reporter assay system kit (Promega, Madison, WI). Relative luciferase activity was calculated by normalizing with the Renilla-luciferase activity.

Human embryonic kidney 293T cells were transfected with reporter constructs of the PGRMC1 promoter, Renilla-luciferase vector and human PPARg-expression vector^61^. The transfected cells were incubated in DMEM containing 10% FBS with or without treatment with 15 µmol l^−1^ TZD for 48 h. Luciferase activities were measured as described above.

### Immunofluorescence staining

3T3L1 cells were fixed in 4% paraformaldehyde and then incubated in 0.1% Tween + PBS solution for 10 min. The cells were then blocked by PBS containing 1% BSA for 30 min. The cells were incubated with primary antibodies at 4 °C overnight and then with a secondary antibody at room temperature for 1 h in the dark. Next, cells were incubated in concanavalin A for the analysis of the endoplasmic reticulum or MITO-ID Red Detection Kit (Enzo Life Sciences, Inc, Farmingdale, NY) for the analysis of the mitochondria. Finally, the cells were incubated in 10 mg l^−1^ DAPI for 15 min, and observed using an LSM710 confocal laser microscope (Carl Zeiss, Jena, Germany).

### Co-immunoprecipitation assays

The expression vector, FLAG-PGRMC1 WT or Y113F, was transfected into 3T3L1 cells by using Lipofectamine 2000 (Life Technologies, Carlsbad, CA). After 48 h, the cells were lysed in NP40 lysis buffer [20 mmol l^−1^ Tris-HCl (pH 7.5), 150 mmol l^−1^ NaCl, 1% NP40], and the lysate was incubated with 10 µl of equilibrated anti-FLAG (M2) agarose for 60 min at room temperature. The bounds proteins were subjected to SDS-PAGE and visualized by western blotting.

For co-immunoprecipitation with endogenous PGRMC1, 3T3L1 cell lysate was incubated with 1 µg anti-PGRMC1 antibody (Abcam: ab48012) or normal rabbit IgG (Abcam: ab37415) for 2 h, and then incubated with 10 µl protein A-sepharose beads (Invitrogen, 101142) for overnight. Precipitates were washed three times in NP40 lysis buffer, and bound proteins were subjected to SDS-PAGE and visualized by western blotting using antibodies against PGRMC1 (Genetex: GTX89362), LDL-R (R&D: AF2255), or GLUT4 (Abcam: ab33780).

### Flow-cytometry analyses

Cells treated with Alexa Fluor 488-acetylated LDL or DiI–VLDL were diluted in PBS containing 1% paraformaldehyde. Thaeman fluorescent intensity per 10,000 cells was analyzed using a flow cytometer (Gallious, Beckman Coulter Life Science, Brea, CA).

### Analyses of fatty acid synthesis using ^13^C_6_-glucose in 3T3L1 cells

Analyses of fatty acid synthesis were performed according to the method by Nagai et al.^62^. Differentiated 3T3L1 WT or PGRMC1-KD cells were incubated with a glucose-free DMEM containing 10% FBS and 4.5 g l^−1^ of [^13^C_6_]-glucose for 24 h. The collected cells were hydrolyzed with 0.5 ml of 20% KOH at 80 °C for 60 min, and later 0.6 ml of 5 N HCl was added. Fatty acids were extracted twice with 0.4 ml diethyl ether, and the extracted solution was dried at 40 °C under N_2._ The fatty acids were dissolved in 0.5 ml of methanol and analyzed by a liquid chromatography–mass spectrometry (LC/MS) system. As an LC/MS system, an orbitrap-type MS (Q-Exactive focus, Thermo Fisher Scientific, San Jose, CA) connected to Thermo Scientific Dionex^-^UltiMate3000 RSLC system was used. As a separation column, Thermo Scientific Accucore C18 (2.1 × 150 mm, 2.6 μm) was used. The LC pump gradient was as follows: 65% mobile phase A (10 mmol l^−1^ HCOONH_4_ in 50% ACN (v) + 0.1% HCOOH (v)) and 35% mobile phase B (2 mmol l^−1^ HCOONH_4_ in ACN/IPA/H_2_O 10:88:2 (v/v/v) + 0.02% HCOOH (v)) from 0 to 4 min, 40% mobile phase A, and 60% mobile phase B from 4.1 to 12 min, 15% mobile phase A and 85% mobile phase B from 12.1 to 21 min, and 100% mobile phase B to 24 min. The injection volume, flow rate, and column temperature were 5 μl, 0.4 mL, and 35 °C, respectively. The Q-Exactive Focus mass spectrometer was operated under an ESI negative mode for all detections. Full mass scan (m/z 250 − 1200) was used at a resolution of 70,000. The automatic gain control target was set at 1 × 10^6^ ions, and the maximum ion injection time was 100 ms. Source ionization parameters were optimized with a spray voltage of 3 kV, and other parameters were as follows: transfer temperature at 285 °C, S-Lens level at 45, heater temperature at 370 °C, Sheath gas at 60, and Aux gas at 20. [^13^C_6_]-glucose, [^13^C_4–12_]-palmitic acid, [^13^C_4–16_]-stearic acid, and [^13^C_4–16_]-oleic acid in cells were measured by LC/MS.

### Animal studies

All the protocols for animal experiments in this study were approved by the Experimental Animal Committee of Keio University School of Medicine [approved number, 08024(10)]. PGRMC1 exon 2 was flanked with loxP sites in C57BL/6J (WT) mice (PGRMC1 *flox/flox* mice). PGRMC1 *flox/flox* mice then were crossed with adiponectin-Cre mice, thereby generating PGRMC1 adipose tissue-specific knockout (AKO) mice. All animal studies were performed at 24 °C.

WT and PGRMC1-AKO male mice at 8–11 weeks of age were fed a normal diet (ND: 4.6% kcal fat; source, soybean; CLEA Japan, Inc., Tokyo, Japan) or a HFD (40% kcal fat; Research Diet, Inc., New Jersey, USA). Body weights and amounts of food ingested by each mouse were measured weekly for 12 weeks. All mice were sacrificed at 16 weeks after the administered diets to collect blood samples and tissues. AST/ALT, TG in serum and liver TG were measured using GPT/GOT C-test and TG E-test (Wako, Osaka, Japan). Total cholesterol, HDL cholesterol, and LDL/VLDL cholesterol in serum were measured using an HDL and LDL/VLDL cholesterol assay kit (Cell Biolab. Inc., San Diego, CA). Serum insulin was measured using an Ultra-Sensitive Mouse Insulin ELISA Kit (Morinaga Institute of Biological Science, Inc., Yokohama, Japan). WAT, BAT, and livers were embedded in paraffin and stained with hematoxylin & eosin. The stained samples were observed using a microscope (ECLIPSE E600, Nikon, Tokyo, Japan). Each adipocyte size was obtained by calculating the average size of 100 adipocytes with Image J software (National Institutes of Health, Maryland, USA).

To analyze the effect of TZD, troglitazone diluted with PBS was administered to mice by an intraperitoneal injection at a dose of 5 mg kg^−1^ body weight on 3 consecutive days, and 1 day later, the mice were sacrificed to collect WAT for analyses of PGRMC1 expression with qPCR or western blotting.

### Glucose tolerance test

Glucose tolerance was examined in WT and PGRMC1-AKO mice 12 weeks after fed either ND or HFD. After fasting for 6 h, glucose was administered intraperitoneally (1.5 mg kg^−1^ weight). Blood glucose levels were measured by a glucometer (ACCUE-CHEK Aviva-Nano, Roche Basel, Switzerland).

### Indirect calorimetry

WT and PGRMC1-AKO mice 12–13 weeks after fed either ND or HFD were acclimatized in metabolic cages for 24 h to the experimental environment before measurements were taken. Mice were kept on 12 h light–12 h dark cycle at 24 °C. Next 24 h, oxygen consumption, respiratory quotient (RQ), heat production, and locomotor activity were measured using the Oxymax System 4.93 (C.L.A.M.S.; Columbus Instruments, Ohio, USA). In addition, spontaneous locomotor activity was measured for 24 h with an OPTO-M3 Activity Application Device (Columbus Instruments, Ohio, USA). We plotted oxygen consumption per mouse in relation to body weight^51^ and evaluated the average of oxygen consumption, RQ, heat production, and locomotor activity.

### Statistical and reproducibility

Experimental results are represented as means ± standard error of means. Student’s *T* test was used for two-group comparisons, and one-way ANOVA with Tukey’s *T* test was used for multiple comparisons. *P* values < 0.05 were considered as significant. No statistical methods were used to predetermine the sample size. The sample size was based on experimental feasibility, sample availability, and N necessary to obtain definitive, significant results. The experiments using cell lines were replicated twice. Animals were randomly assigned among the various groups.

### Reporting summary

Further information on research design is available in the Nature Research Reporting Summary linked to this article.

## Supplementary information

Supplemental Information

Description of Additional Supplementary Files

Supplementary Data 1

Reporting Summary

## Data Availability

All data supporting the findings of this study are available within the paper and its Supplementary Files or available from the corresponding author upon reasonable request. Source data underlying plots shown in figures are available in Supplementary Data 1. The full, uncropped images of western blots are shown in Supplementary Fig. 17.
