# Supplementary material for: Progesterone receptor membrane associated component 1 enhances obesity progression in mice by facilitating lipid accumulation in adipocytes
Source: Commun Biol. 2020 Sep 4;3:479. doi: 10.1038/s42003-020-01202-x (PMC7473863; doi:10.1038/s42003-020-01202-x)
Supplement: Supplementary file 1 — Supplemental Information [file 42003_2020_1202_MOESM1_ESM.docx]

**Supplemental information**

**Progesterone receptor membrane associated component 1 regulates obesity progression by regulating lipid accumulation in adipocytes**

**
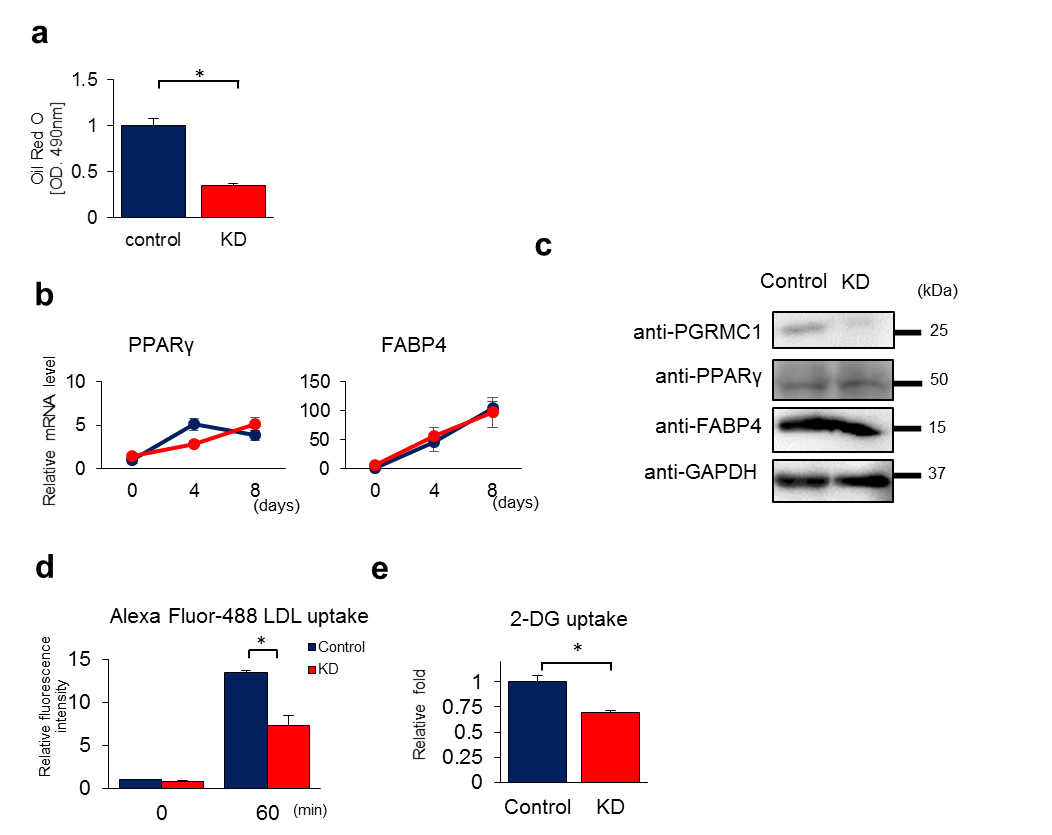
Supplementary Figure 1**

**Supplementary Figure 1 PGRMC1 contributes to lipid accumulation on 3T3L1 differentiation induced by TZD.**

**(a)** Oil red O staining of differentiated 3T3L1 cells treated with TZD. 3T3L1 cells (Control or PGRMC1KD) were differentiated with treated of 15 μmol l^-1^ TZD every 2 days, and the cells were stained with Oil red O. The graph depicts the absorbance at 490 nm by Oil red O (n=12).  **(b)** Analyses of mRNA expression of *PPARγ or FABP4* in 3T3L1 (Control, KD) cells treated with TZD (n=5). The graph shows relative fold change by normalizing with mRNA levels in Control cells at day 0. **(c)** Analyses of protein expressions in differentiated 3T3L1 cells (Control, KD) treated with TZD by western blotting using antibodies against PGRMC1, PPARγ, FABP4 or GAPDH. (**d**) Flowcytometric analysis of fluorescence intensities of differentiated 3T3L1 cells (Control, KD) treated with TZD after incubation with Alexa Fluor 488 acetylated LDL for 60 min. The graph shows the mean fluorescence intensities (per 10,000 cells) (n = 5). (**e**) Analysis of the effect on insulin-stimulated 2-DG uptake. After treatment with 0.5 μmol l^-1^ insulin for 18 min, differentiated 3T3L1 cells (Control, KD) treated with TZD were incubated with 1 μmol l^-1^ 2-DG for 20 min, and the 2-DG uptake was measured. The graph shows relative fold change by normalizing with 2-DG uptake of 3T3L1 control cells (n = 5). Data are represented as mean ± S.E. Statistical analysis was performed using Student’s T test (**a**, **d** and **e**) or ANOVA with Tukey’s T test (**b**). ^*^P < 0.05

**Supplementary Figure 2
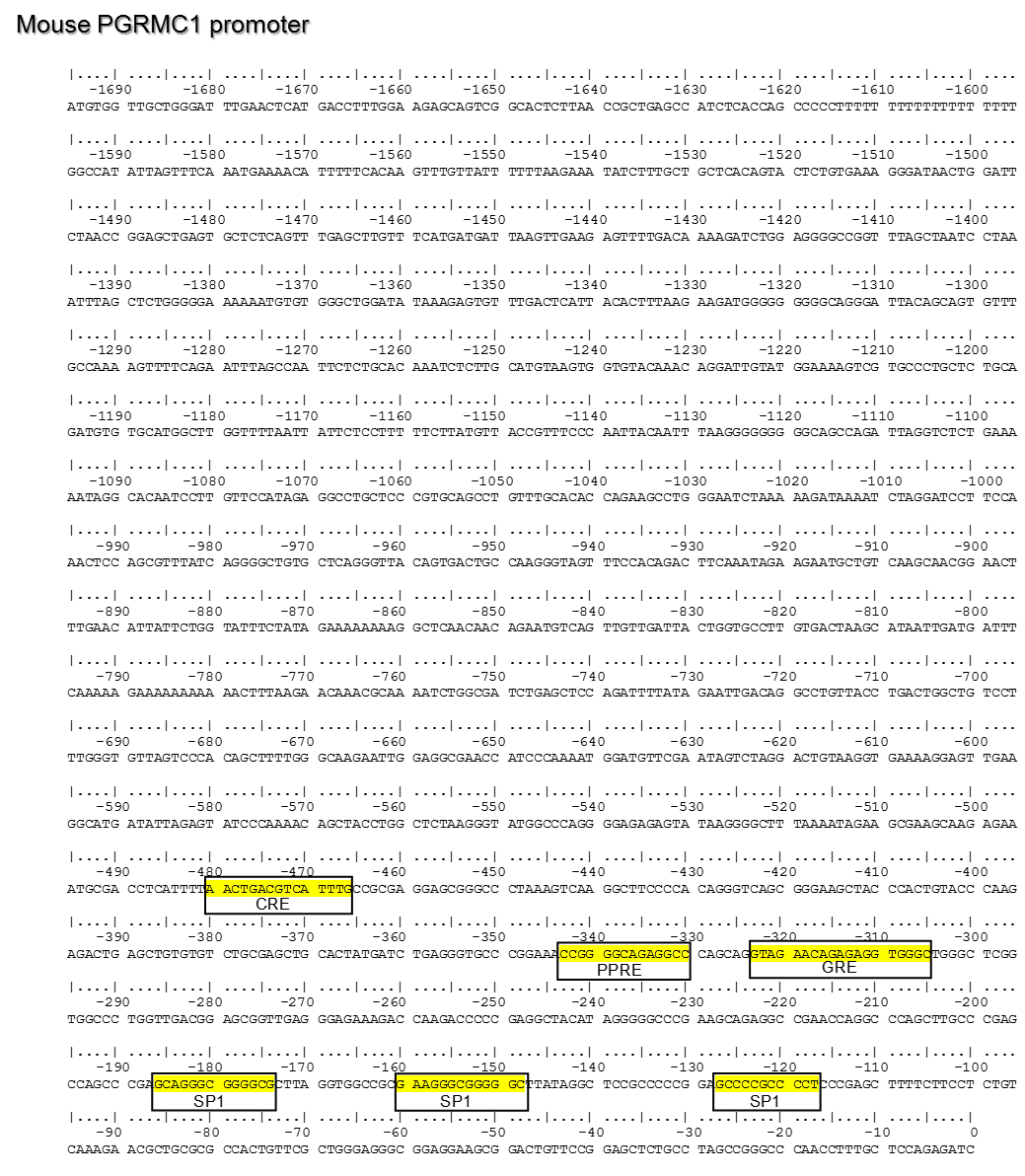
**

**Supplementary Figure 2 Prediction of transcription factor binding sites in mouse *PGRMC1* promoter sequence.**

Transcription factor binding sites in mouse *PGRMC1* promoter sequence were predicted using the TRANSFAC software.

**
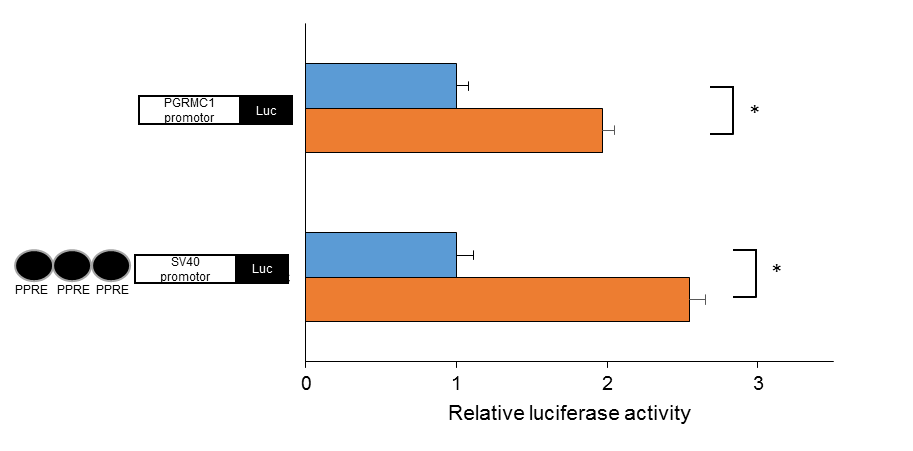
Supplementary Figure 3**

**Supplementary Figure 3 TZD enhances the *PGRMC1* promoter activity in 293 cells.**

Reporter gene assay of mouse *PGRMC1* promoter in 293T cells by stimulation with TZD. The reporter constructs of the PGRMC1 promoter (-1695/+1-PGRMC1-Luc) or the PPRE x3-SV40 promoter containing construct were transfected into 3T3L1 cells and then incubated with 15 μmol l^-1^ TZD for 2 days. The graph shows relative luciferase activity after normalizing with luciferase activity in 293T cells without treatment by TZD (n = 3). All data are represented as mean ± S.E. Statistical analysis was performed with Student’s T test. ^*^P < 0.05.

**
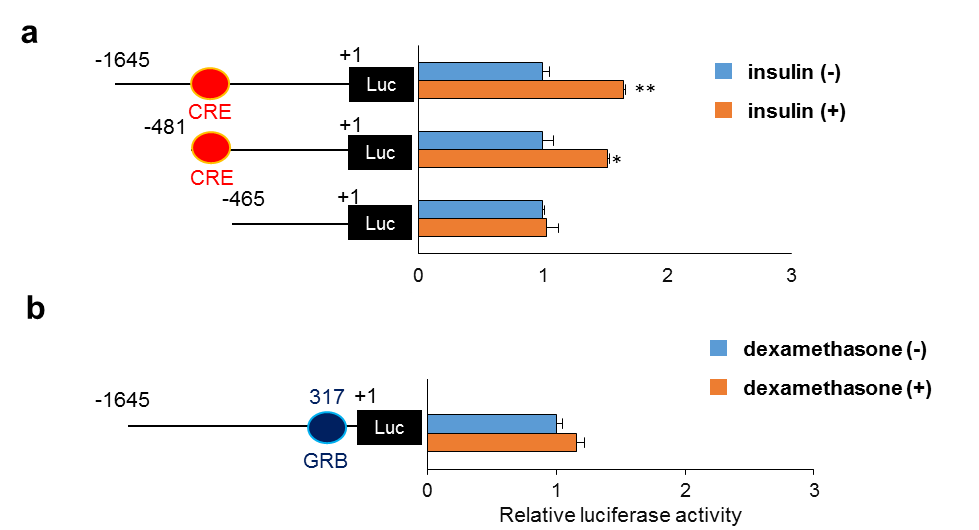
Supplementary Figure 4**

**Supplementary Figure 4 Insulin enhances the *PGRMC1* promoter activity.**

**(a)** Reporter gene assay of mouse *PGRMC1* promoter by stimulation with insulin. The reporter constructs of PGRMC1 promoter containing ATF/CREB sequences (−1695/+1-PGRMC1-Luc, -681/+1-PGRMC1-Luc) or lacking ATF/CREB sites (-665/+1-PGRMC1-Luc) were transfected into 3T3L1 cells and then incubated with 0.5 μmol l^-1^ insulin for 2 days. The graph shows relative luciferase activity after normalizing with luciferase activity in 3T3L1 cells without treatment by insulin (n = 3). **(b)** Reporter gene assay of mouse PGRMC1 promoter. The reporter constructs of *PGRMC1* promoter containing GR sequences (−1695/+1-PGRMC1-Luc) was transfected into 3T3L1 cells, and the cells were incubated for 2 days after adding 1 μmol l^-1^ dexamethasone. The graph shows relative luciferase activity after normalizing with luciferase activity in 3T3L1 cells without treatment with dexamethasone (n = 3). All data are represented as mean ± S.E. Statistical analysis was performed with Student’s T test. ^*^P < 0.05, ^**^P < 0.01.

**
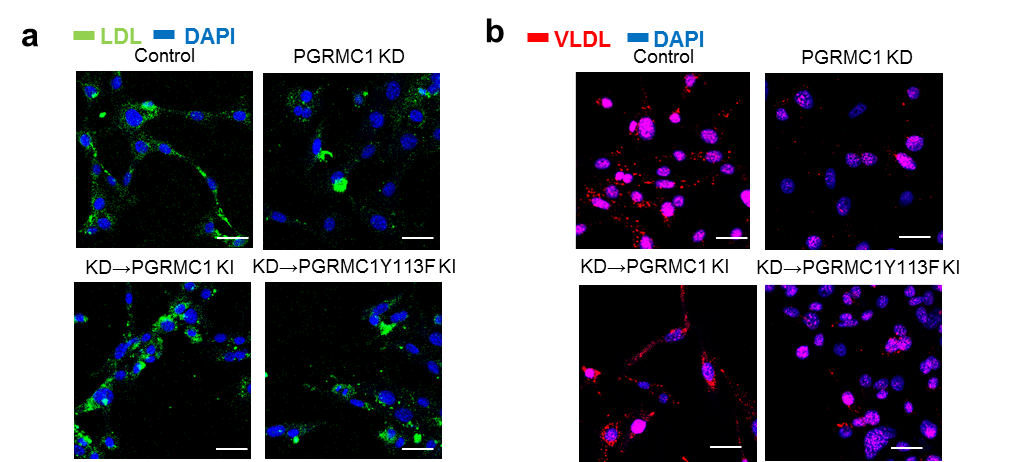
Supplementary Figure 5**

**Supplementary Figure 5 PGRMC1 contributed to LDL and VLDL uptake.**

The fluorescent-stained cells images of different area of Fig. **3a** and **3c**. (**a**) The images show 3T3L1 control cells, PGRMC1 KD cells, or PGRMC1 KD cells expressing shRNA-resistant PGRMC1-WT or PGRMC1-Y113F stained with Alexa Fluor 488 acetylated LDL (green) and DAPI (blue) (Scale bar; 10 μm). (**b**) the images show control cells, PGRMC1 KD cells, or PGRMC1 KD cells expressing shRNA-resistant PGRMC1-WT or PGRMC1-Y113F stained with DiI-VLDL (red) and DAPI (blue) (Scale bar; 10 μm).

**
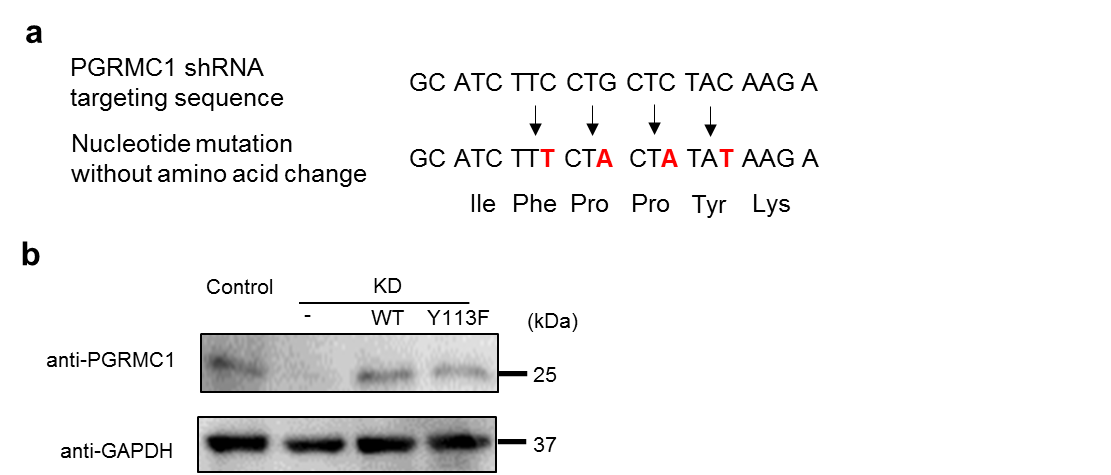
 Supplementary Figure 6**

**Supplementary Figure 6 Rescue experiments of PGRMC1 expression resistant to the knockdown.**

(**a**) Nucleotide sequences of *PGRMC1* targeted by shRNA (upper panel). Construction of the shRNA-resistant mutated *PGRMC1* sequences without amino acid changes (lower panel). (**b**) Stable PGRMC1-KD 3T3L1 cells were transiently transfected with the shRNA-resistant expression vector of wild type PGRMC1 (WT) or the Y113F mutant (Y113F). The protein expressions were analyzed by western blotting using antibodies against PGRMC1 or GAPDH.

**
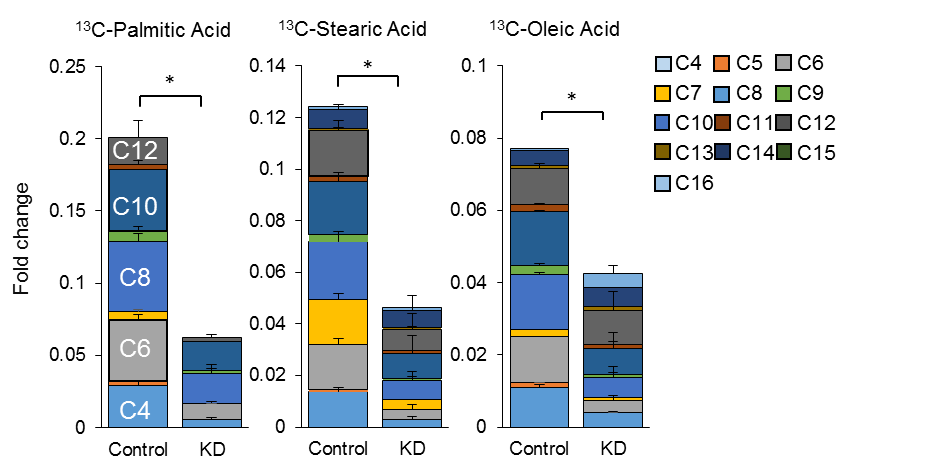
Supplementary Figure 7**

**Supplementary Figure 7 Analyses of the effect of fatty acid synthesis by PGRMC1 using [^13^C_6_]-glucose.**

After differentiated 3T3L1 cells (Control and KD) were incubated with 4.5 g l^-1^ [^13^C_6_]-glucose for 24 h, the fatty acids were extracted. Labeled fatty acids i.e., [^13^C_4-12_]-palmitic acid, [^13^C_4-16_]-stearic acid and [^13^C_4-16_]-oleic acid in cells were measured by LC/MS and each of fractions were shown. (n = 3).

**Supplementary Figure 8**

**
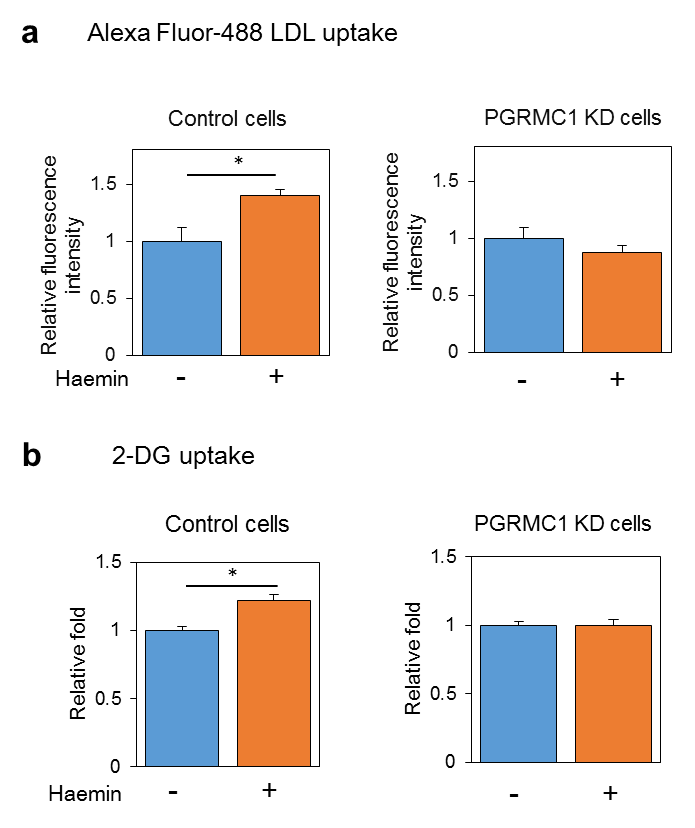
**

**Supplementary Figure 8 Haemin enhances the LDL uptake and 2-DG uptake in adipocyte.**

(**a**) Analysis of the effect to LDL uptake by haemin. Control or PGRMC1 KD 3T3L1 cells were incubated with or without 10 μmol l^-1^ haemin for 1 hour, then incubated with Alexa Fluor 488 acetylated LDL for 1 hour. The graph shows the mean of fluorescence intensities (per 10,000 cells) (n = 4-5) **(b)** Analysis of the effect to 2-DG uptake by haemin. After treatment of 0.5 μmol l^-1^ insulin for 18 min with or without 10 μmol l^-1^ haemin for 1 hour, 3T3L1 cells were incubated with 1 μmol l^-1^ 2-DG for 20 min, and the 2-DG uptake was measured. The graph shows relative fold change by normalizing with 2-DG uptake of 3T3L1 cells without treatment of haemin (n = 3). Data are represented as mean ± S.E. Statistical analysis was performed using Student’s T test. ^*^P < 0.05

**
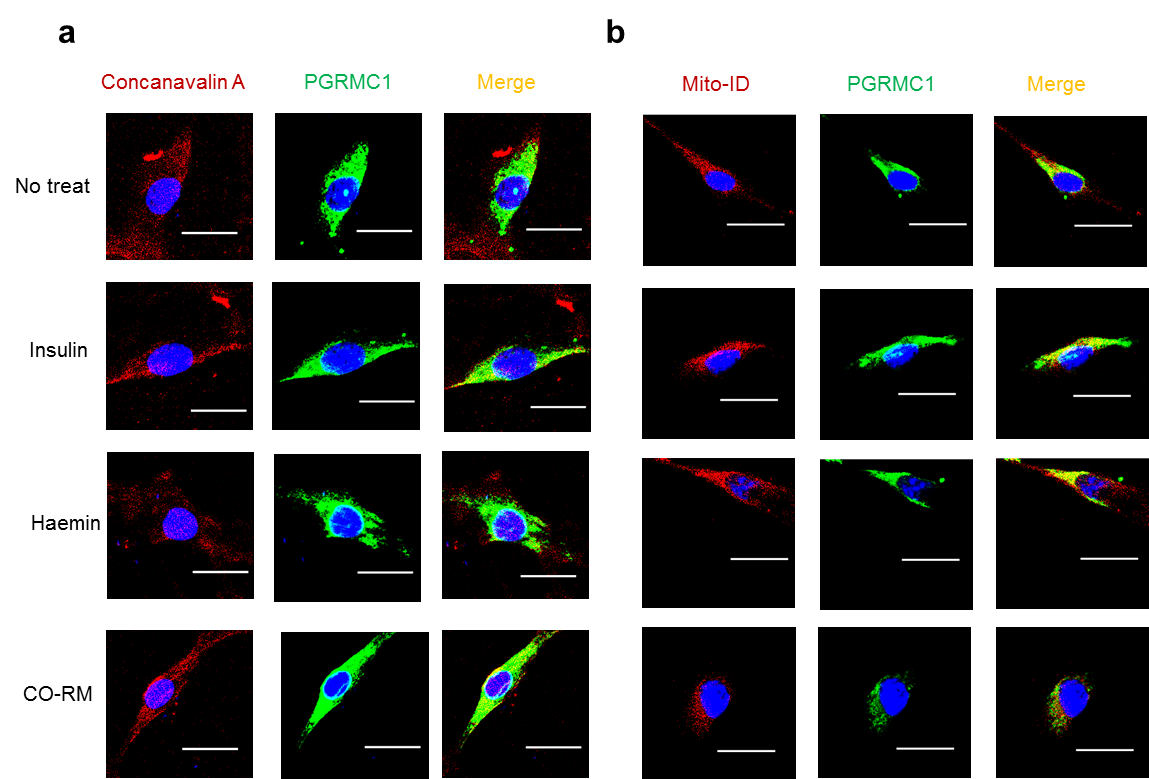
Supplementary Figure 9**

**Supplementary Figure 9 Cellular localization of PGRMC1 in 3T3L1.**

Confocal microscopic image of 3T3L1 control cells or those treated with 0.5 μmol l^-1^ insulin for 18 min**,** 10 μmol l^-1^ CO-RM for 2 hours or 10 μmol l^-1^ haemin for 1 hour. **(a)** The left images show the localization of PGRMC1 (green fluorescence), Concanavalin A (red fluorescence) and DAPI (blue fluorescence). **(b)** The right images show the localization of PGRMC1 (green fluorescence), Mito-ID (red fluorescence) and DAPI (blue fluorescence).

**
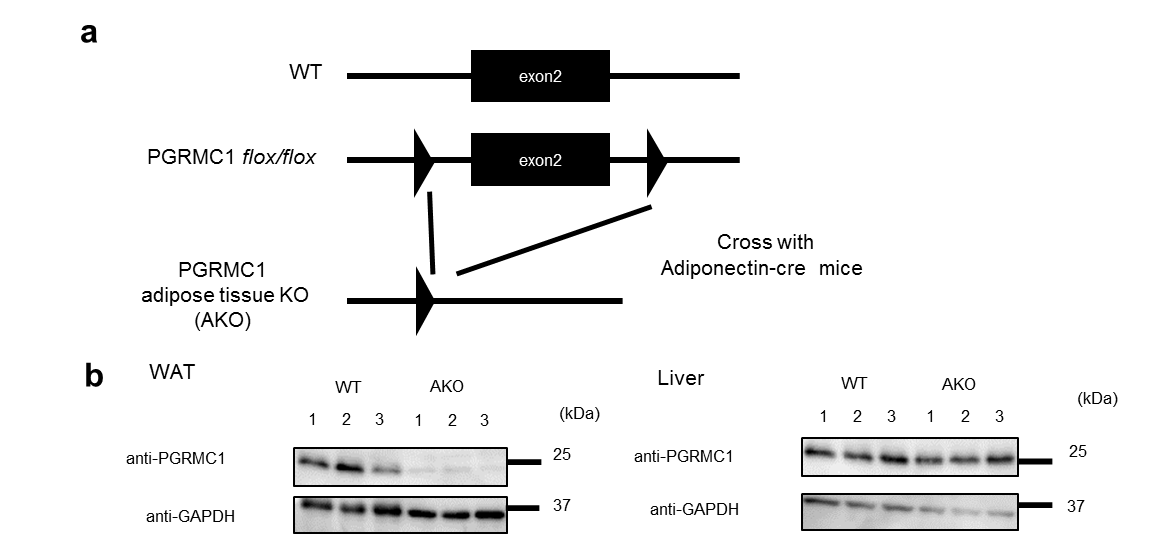
Supplementary Figure 10**

**Supplementary Figure 10 Generation of PGRMC1 AKO mice.**

**(a)** Scheme of the generation of PGRMC1 adipose tissue knockout (AKO) mice. PGRMC1 flox/flox mice was generated by insertion of 2 flox sites across the PGRMC1 exon 2 in C57BL/6J (WT) mice, and crossed with Adiponectin-Cre mice to create PGRMC1 AKO mice. **(b)** Analyses of protein expressions in white adipose tissue (WAT) and liver of WT or PGRMC1 AKO mice by western blotting using antibodies against PGRMC1 or GAPDH.

**
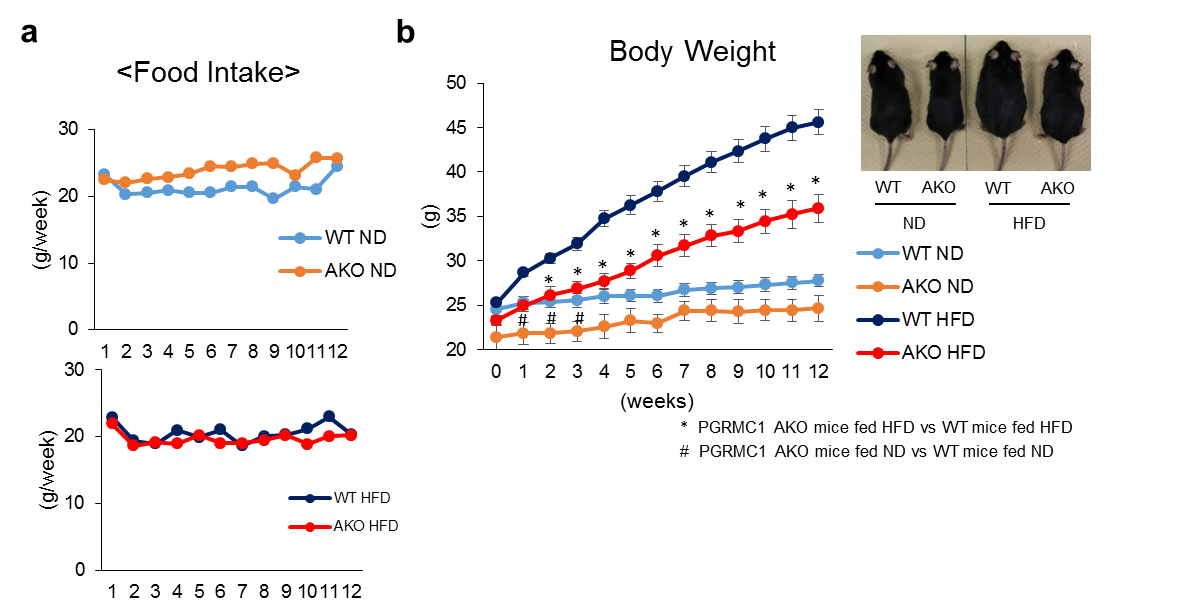
Supplementary Figure 11**

**Supplementary Figure 11 Adipose tissue-specific knockout of PGRMC1 expression suppresses the body weight increase by high fat diet.**

**(a)** Food intake in WT mice and PGRMC1 AKO mice fed HFD (n = 8). (**b**) Body weight changes in WT mice and PGRMC1 AKO mice fed ND or HFD for 12 weeks. (n = 5-8). Photographs of these mice after feeding are shown in the right panel. Data are represented as mean ± S.E. Statistical analysis was performed using ANOVA with Tukey’s T test. ^*^P, ^#^P < 0.05.

**Supplementary Figure 12**

**
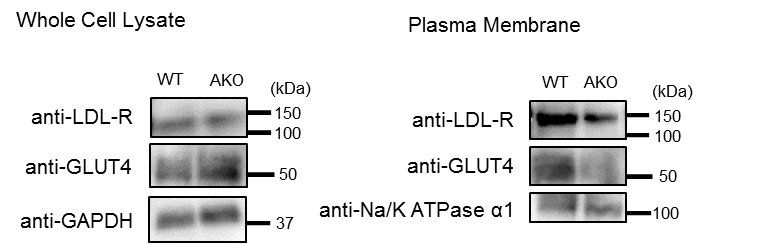
**

**Supplementary Figure 12 PGRMC1 contributes to regulate the translocation to the plasma membrane of LDL-R or GLUT4 in white adipose tissue.**

Analyses of regulation of the LDL-R or GLUT4 translocation by PGRMC1. Plasma membrane fractions were extracted from white adipose tissue (WT mice or PGRMC1 AKO mice fed HFD). The plasma membrane proteins or whole cell lysates were detected by western blotting using antibodies against LDL-R, GLUT4, GAPDH and Na-K ATPase α1.

**
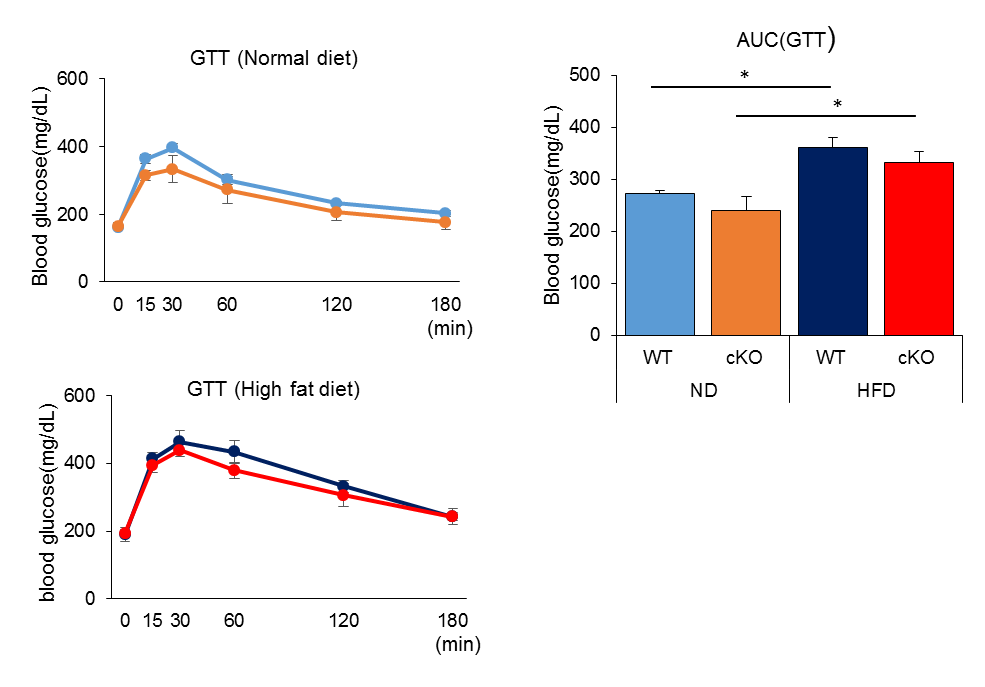
Supplementary Figure 13**

**Supplementary Figure 13 Analyses of the effects in glucose tolerance by PGRMC1 AKO.**

Glucose was administered intraperitoneally (1.5 mg/kg weight) in WT mice or PGRMC1 AKO mice 12 weeks after fed normal diet (ND) or high fat diet (HFD). The graph on the left shows blood glucose curve, and the right one shows the average area under the blood glucose curve (AUC) in WT and PGRMC1 AKO mice fed ND and HFD (n = 5-8). Data are represented as mean ± S.E. Statistical analysis was performed using ANOVA with Tukey’s T test. ^*^P < 0.05

**Supplementary Figure 14**


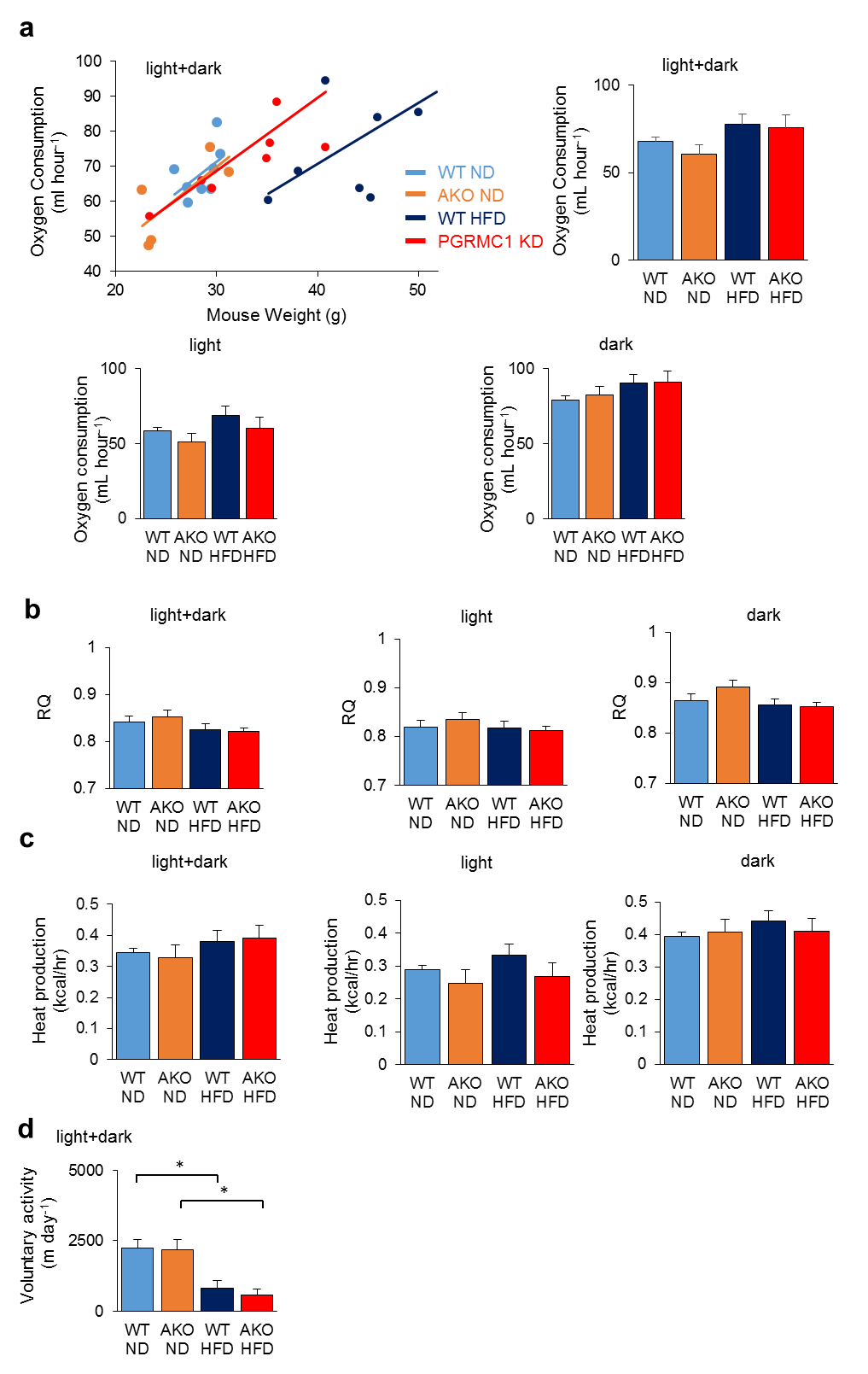


**Supplementary Figure 14 Analyses of the effects in energy expenditure and voluntary activity by PGRMC1 AKO.**

(**a**) Oxygen consumption of WT and PGRMC1 AKO mice fed ND or HFD. The graph on the upper left shows regression plot comparing the oxygen consumption as a function of mouse weight, the average of oxygen consumption in WT and PGRMC1 AKO mice 12-13 weeks after fed ND or HFD (n = 5-8). The upper right graph shows the average of oxygen consumption for 24 hours (light+dark period). The lower graphs show the average of oxygen consumption for 12h light period (left) or 12h dark period (right). (**b**) Respiratory quotient (RQ) of WT and PGRMC1 AKO mice fed ND or HFD for 24 h (left), 12h light period (middle) or 12h dark period (right). (n = 5-8). (**c**) Heat production of WT and PGRMC1 AKO mice fed ND or HFD for 24 h (left), 12h light period (middle) or 12h dark period (right). (n = 5-8). (**d**) Voluntary activity of WT and PGRMC1 AKO mice fed ND or HFD Data are represented as mean ± S.E. Statistical analysis was performed using ANOVA with Tukey’s T test. ^*^P<0.05.

**
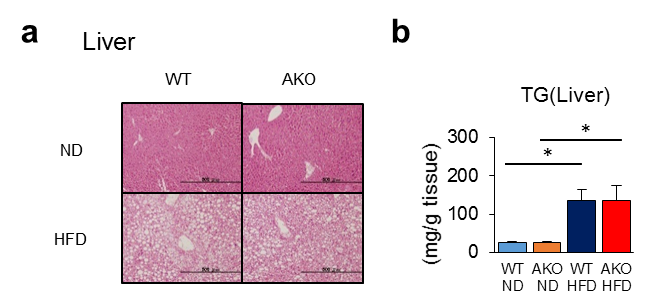
Supplementary Figure 15**

**Supplementary Figure 15 Analyses of the effects in BAT and liver by PGRMC1 AKO.**

**(a)** The paraffin sections of liver in WT and PGRMC1 AKO mice fed ND and HFD stained with hematoxylin and eosin (Scale bar; 500 μm). **(b)** Intrahepatic triglyceride (TG) level of WT and PGRMC1 AKO mice fed ND and HFD (n = 5-8). Data are represented as mean ± S.E. Statistical analysis was performed using ANOVA with Tukey’s T test. ^*^P<0.05.

**
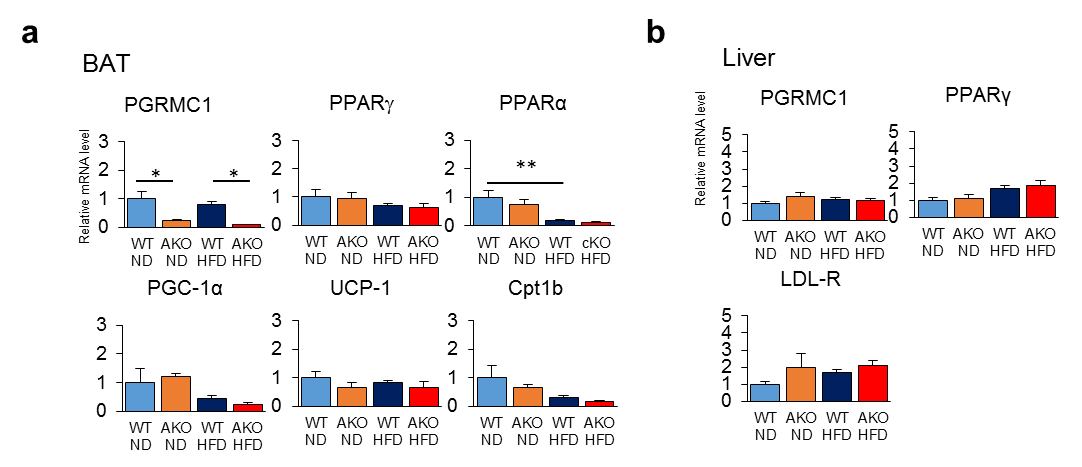
Supplementary Figure 16**

**Supplementary Figure 16 Analyses of the effects in BAT and liver by PGRMC1 AKO.**

**(a)** Analyses of mRNA expressions of *PGRMC1*, *PPARγ*, *PPARα*, *PGC1-α* ,*Cpt-1b*, *and UCP-1* in BAT of WT or PGRMC1 AKO mice fed ND or HFD by qPCR (n = 5-8). The graph shows relative fold change by normalizing with mRNA level of GAPDH. **(b)** Analyses of mRNA expressions of *PGRMC1*, *PPARγ*, *and LDL-R* in the liver of WT or PGRMC1 AKO mice fed ND or HFD by qPCR (n = 5-8). The graph shows relative fold change after normalizing with mRNA level of GAPDH. Data are represented as mean ± S.E. Statistical analysis was performed using ANOVA with Tukey’s T test. ^*^P<0.05, ^**^P<0.01.


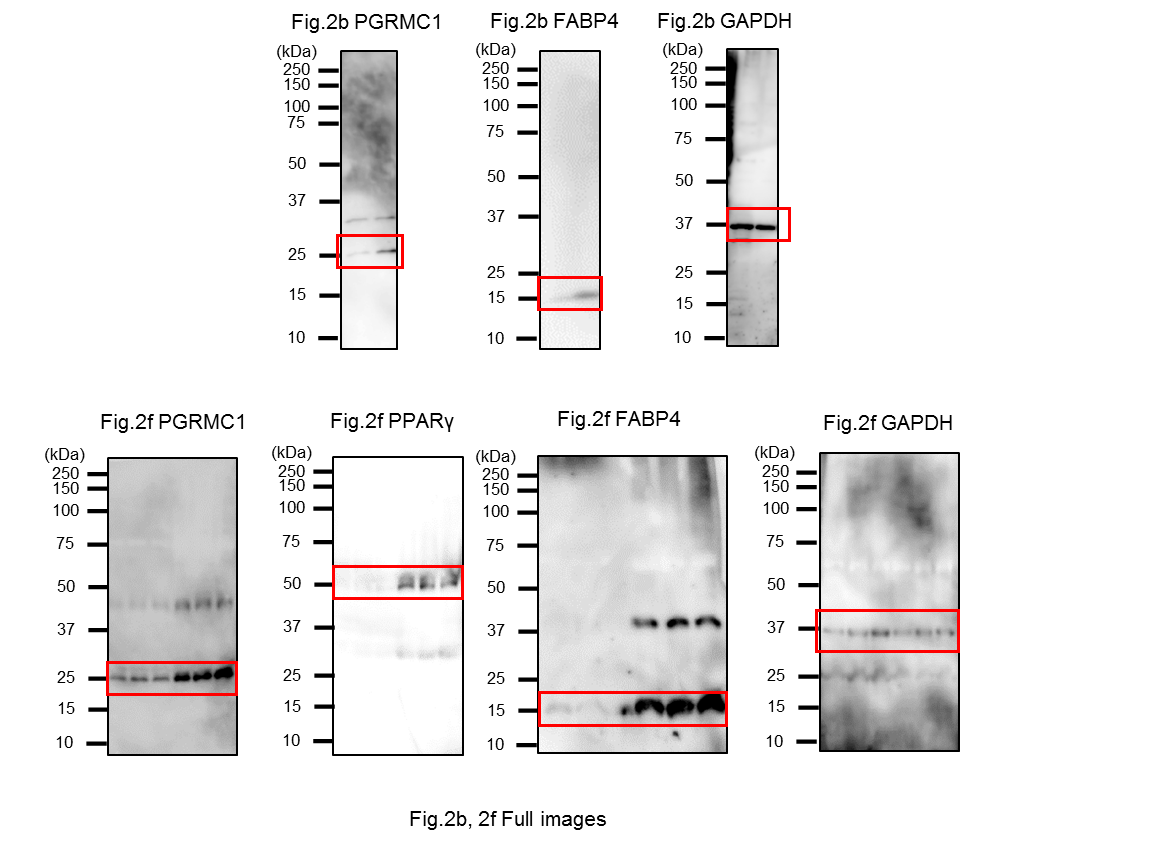
**
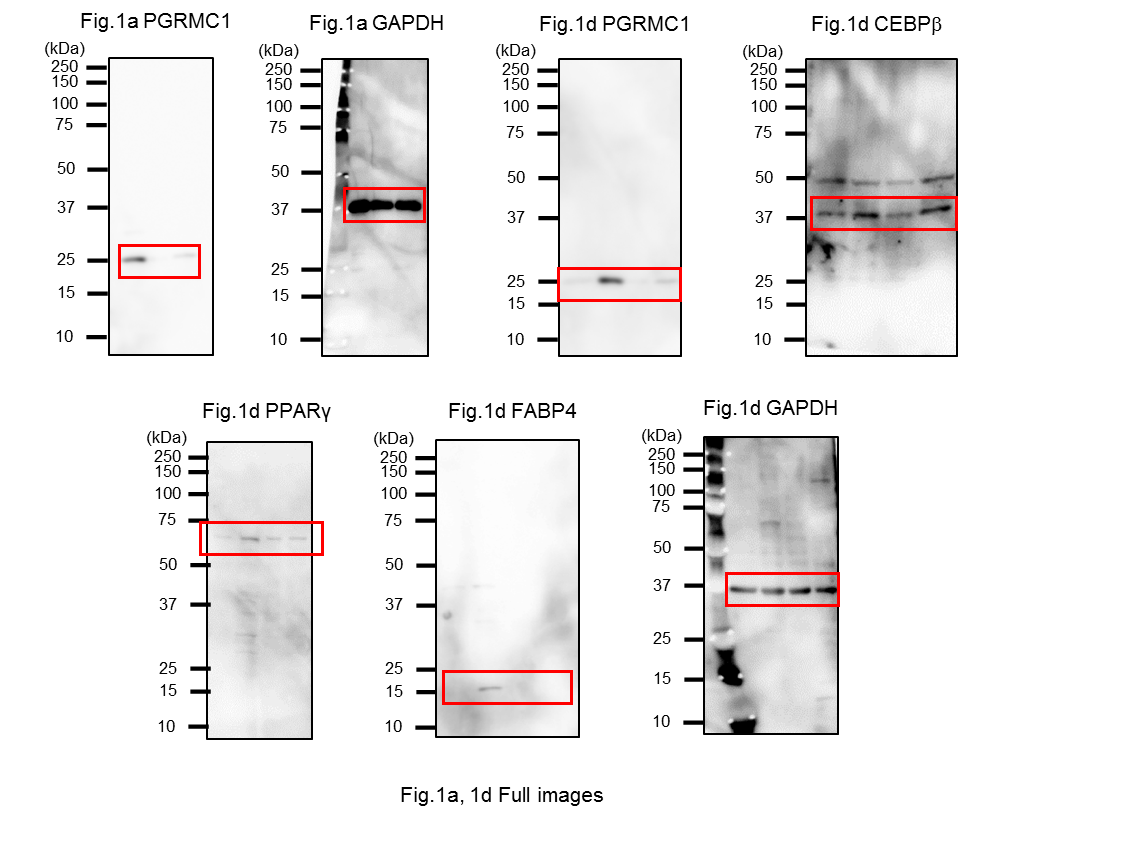
Supplementary Figure 17**


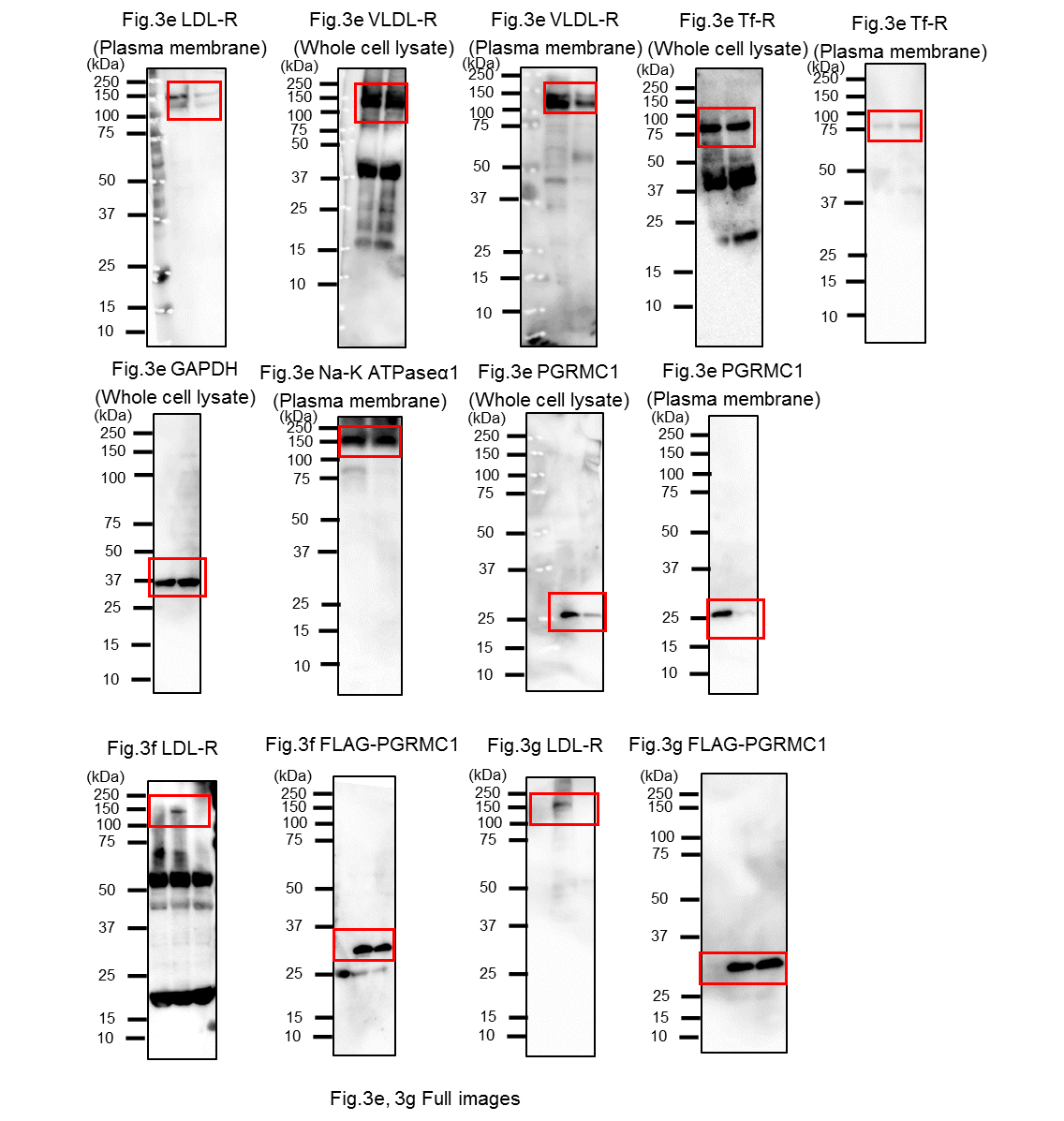


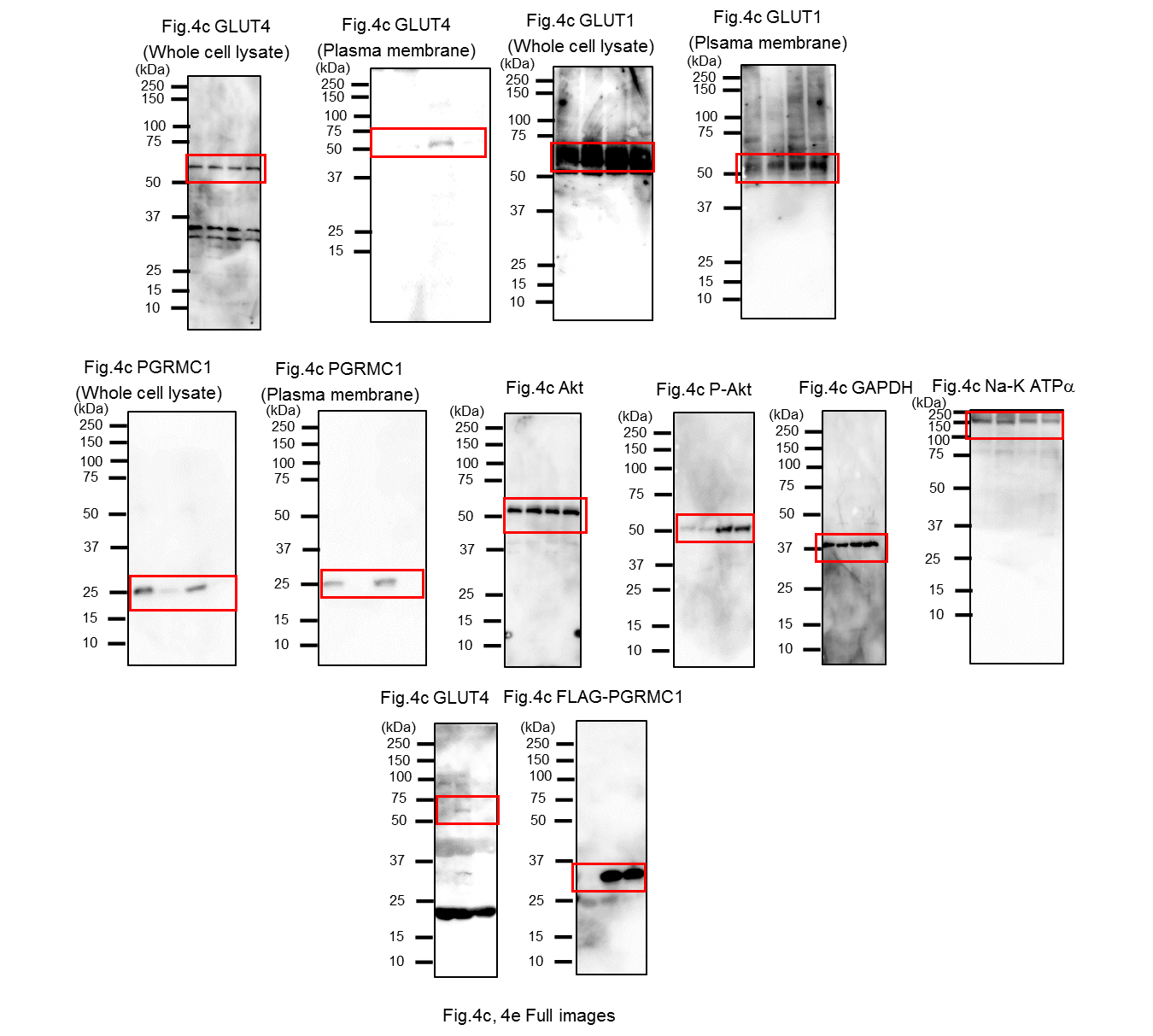


**
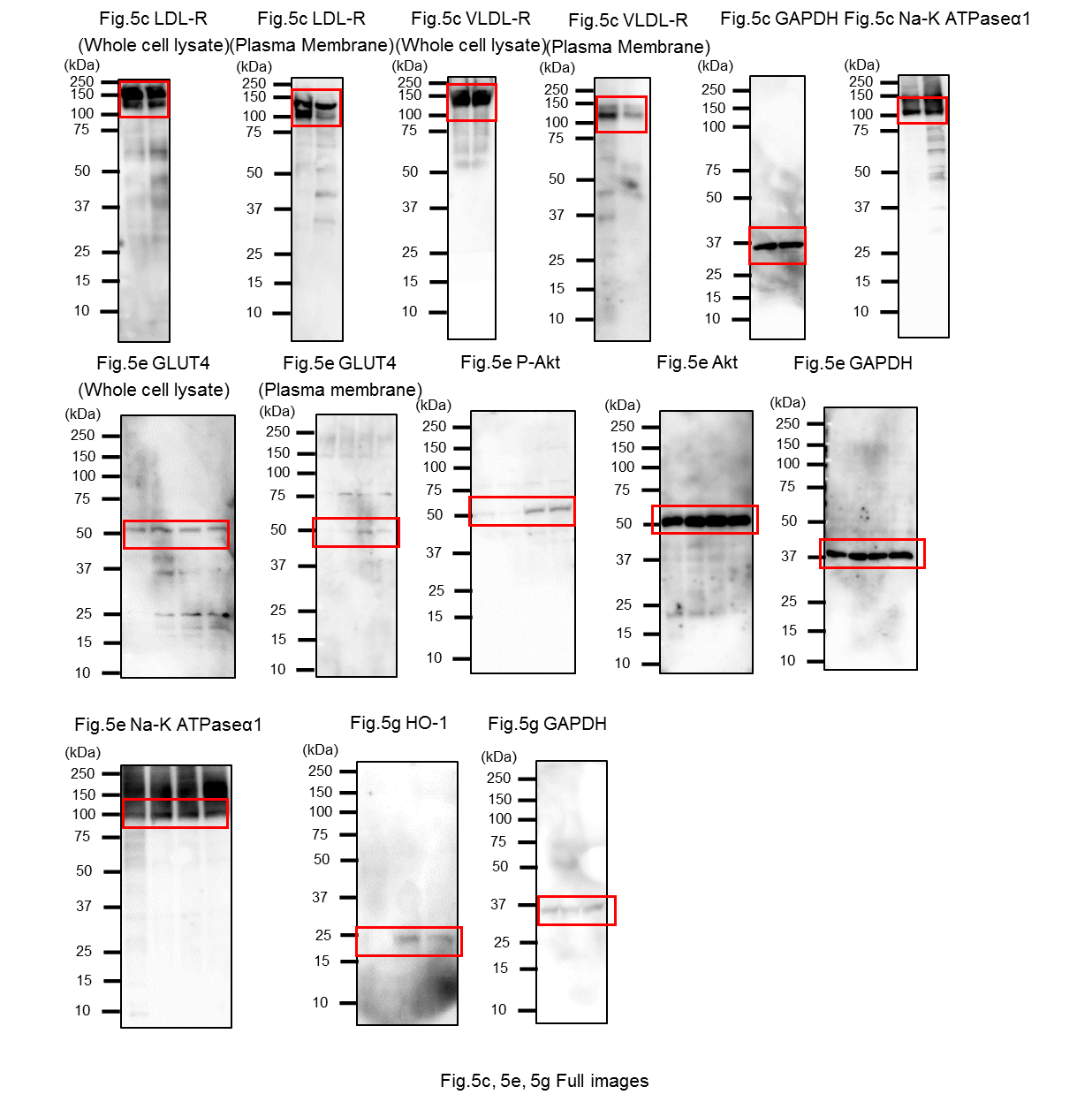
**

**
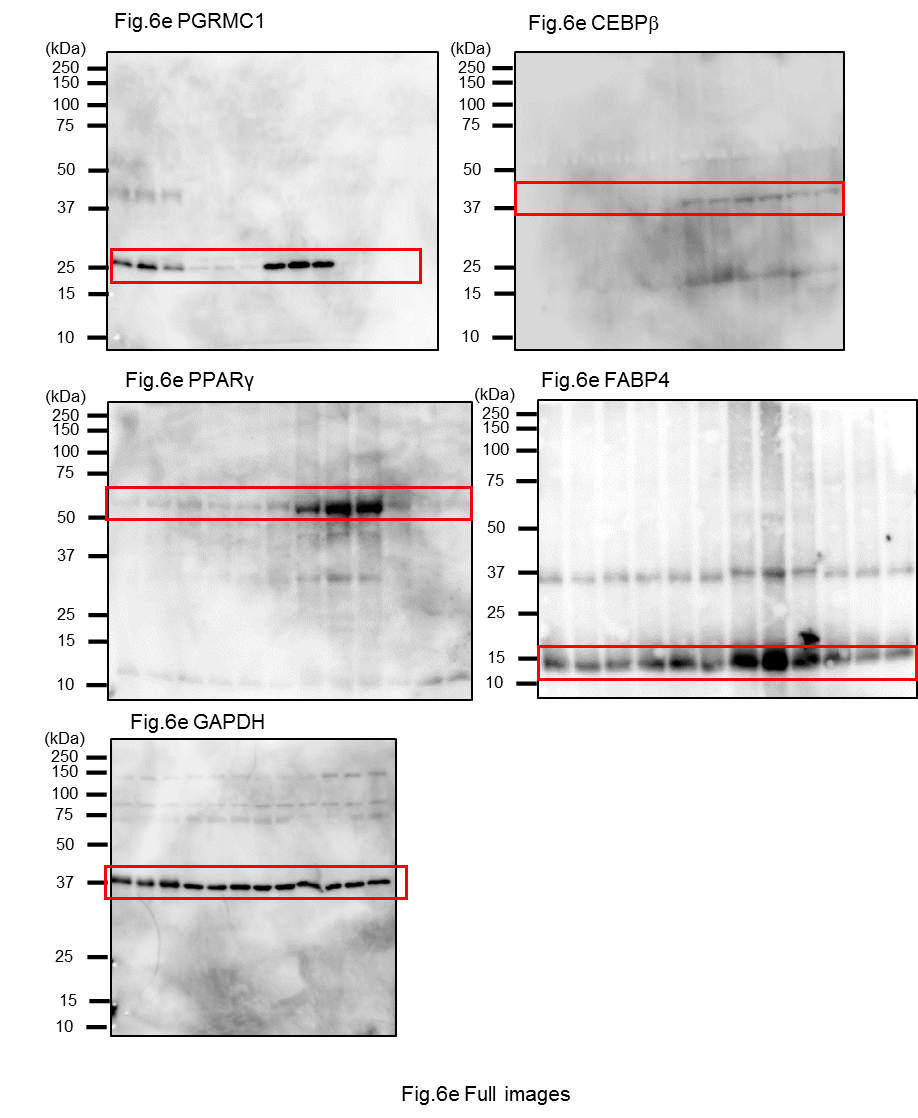
**

**
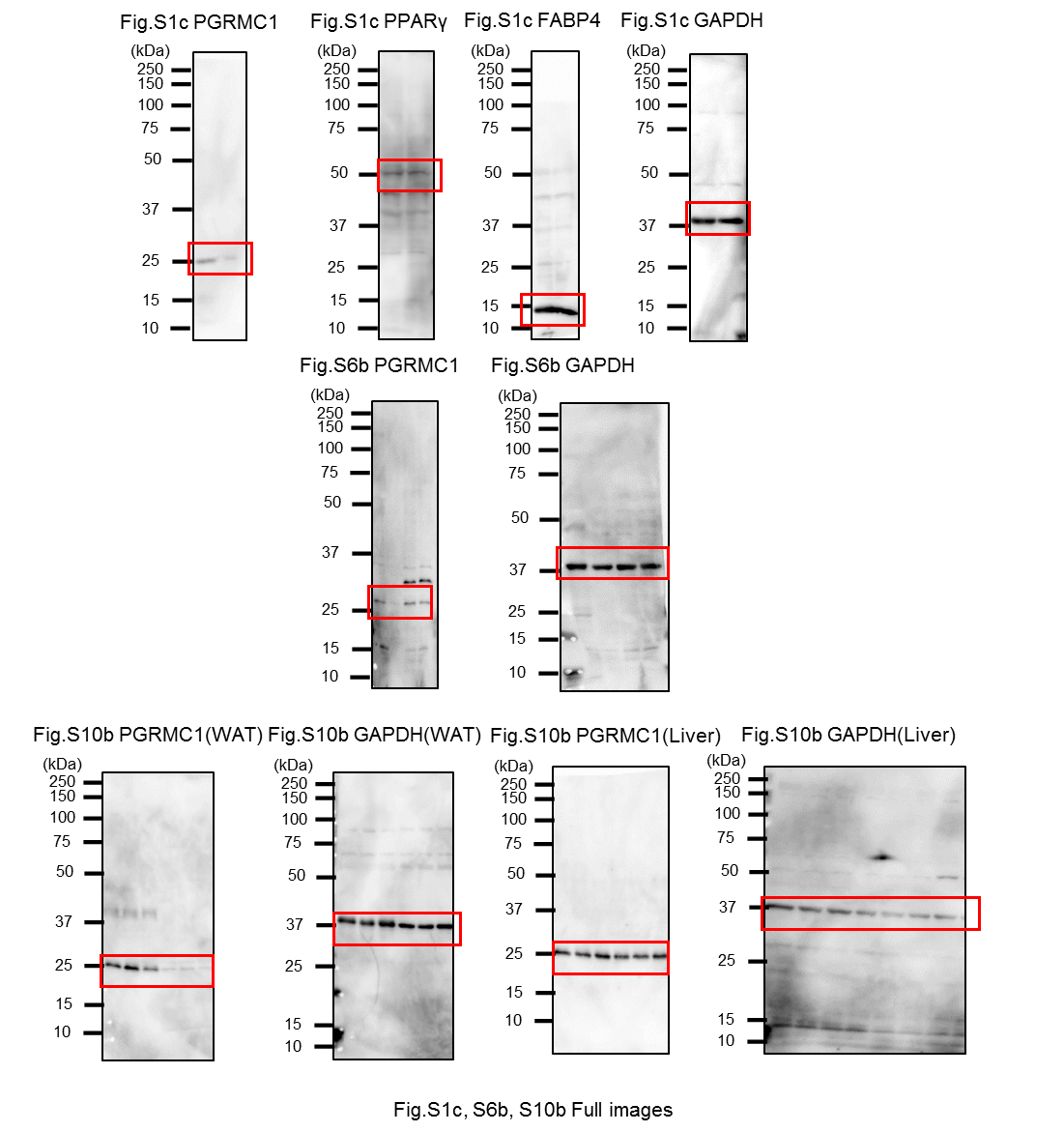
**

**
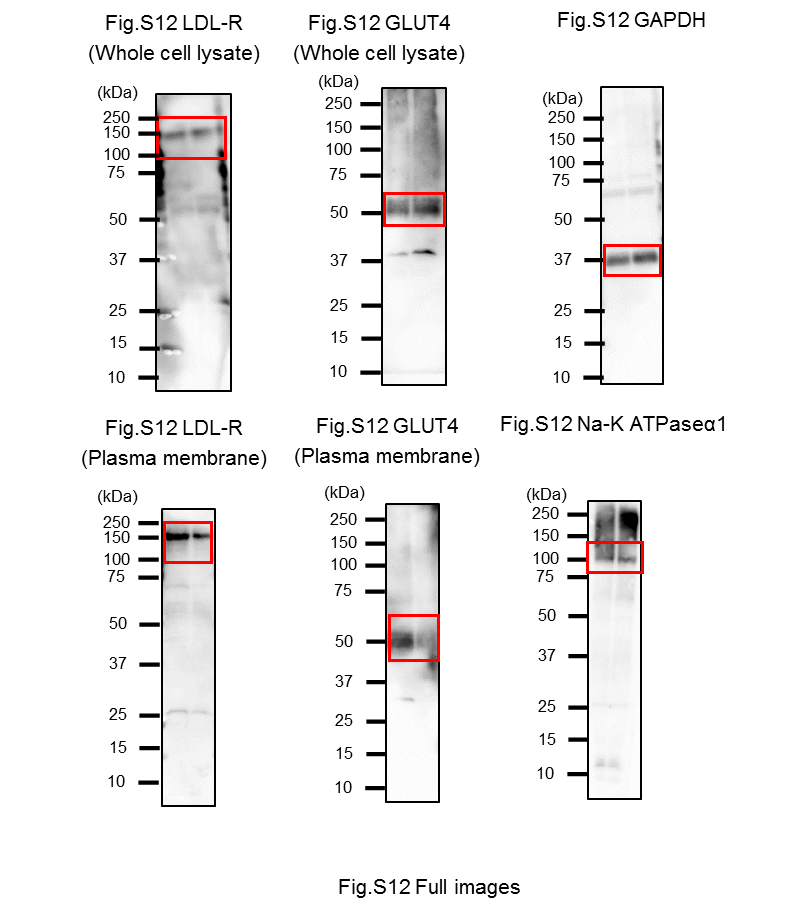
**

**Supplementary Figure 17 Full images of Western blots**

**Supplementary Table 1** **Primer**

| Gene name | Forward Primer 5’-3’ | Reverse Primer 5’-3’ |
| --- | --- | --- |
| mGAPDH | ACCAGGGCTGCATTTGCAGTGGC | TTCACACCCATCACAAACATGGG |
| mPGRMC1 | CTGCTGACGAGATTTTCACGTC | GGCCCCTGGATGCATCTCTTCCG |
| mPPARγ | CATTCACAAGAGCTGACCCAATGG | ACTTCTGAAACCGACAGTACTGAC |
| mFABP4 | AATGTGTGATGCCTTTGTGG | AATTTCCATCCAGGCCTCTT |
| mLDL-R | GTAGAGACGGAAAATGCATCG | CAATCTCGGTCTCCATCACACAC |
| mUCP-1 | TGCCTGGCAGATATCATCAC | CAGTTTCGGCAATCCTTCTG |
| mCPT1β | AGGCCTCGATGACAAGAATG | TTCCGGAAGAGATCTTGGAG |
| mPGC-1α | AAGAGCGCCGTGTGATTTAC | CCATCATCCCGCAGATTTAC |
| mPPARα | AATGCAATTCGCTTTGGAAG | GGCCTTGACCTTGTTCATGT |
