# Supplementary material for: Progesterone receptor membrane associated component 1 enhances obesity progression in mice by facilitating lipid accumulation in adipocytes
Source: Commun Biol. 2020 Sep 4;3:479. doi: 10.1038/s42003-020-01202-x (PMC7473863; doi:10.1038/s42003-020-01202-x)
Supplement: Supplementary file 2 — Description of Additional Supplementary Files [file 42003_2020_1202_MOESM2_ESM.pdf]

Description of Additional Supplementary Files

File Name: **Supplementary Data 1**
Description: source data file
