# Supplementary material for: Progesterone receptor membrane associated component 1 enhances obesity progression in mice by facilitating lipid accumulation in adipocytes
Source: Commun Biol. 2020 Sep 4;3:479. doi: 10.1038/s42003-020-01202-x (PMC7473863; doi:10.1038/s42003-020-01202-x)
Supplement: Supplementary file 4 — Reporting Summary [file 42003_2020_1202_MOESM4_ESM.pdf]

nature research

Corresponding author(s): Yasuaki Kabe

Last updated by author(s): Jul 21, 2020

Reporting Summary

Nature Research wishes to improve the reproducibility of the work that we publish. This form provides structure for consistency and transparency in reporting. For further information on Nature Research policies, see Authors & Referees and the Editorial Policy Checklist.

Statistics

For all statistical analyses, confirm that the following items are present in the figure legend, table legend, main text, or Methods section.

n/a	Confirmed	
☐	☒	The exact sample size (*n*) for each experimental group/condition, given as a discrete number and unit of measurement
☐	☒	A statement on whether measurements were taken from distinct samples or whether the same sample was measured repeatedly
☐	☒	The statistical test(s) used AND whether they are one- or two-sided<br>*Only common tests should be described solely by name; describe more complex techniques in the Methods section.*
☐	☒	A description of all covariates tested
☐	☒	A description of any assumptions or corrections, such as tests of normality and adjustment for multiple comparisons
☐	☒	A full description of the statistical parameters including central tendency (e.g. means) or other basic estimates (e.g. regression coefficient) AND variation (e.g. standard deviation) or associated estimates of uncertainty (e.g. confidence intervals)
☐	☒	For null hypothesis testing, the test statistic (e.g. *F*, *t*, *r*) with confidence intervals, effect sizes, degrees of freedom and *P* value noted<br>*Give P values as exact values whenever suitable.*
☒	☐	For Bayesian analysis, information on the choice of priors and Markov chain Monte Carlo settings
☒	☐	For hierarchical and complex designs, identification of the appropriate level for tests and full reporting of outcomes
☒	☐	Estimates of effect sizes (e.g. Cohen's *d*, Pearson's *r*), indicating how they were calculated

Our web collection on statistics for biologists contains articles on many of the points above.

Software and code

Policy information about availability of computer code

Data collection	No software was used.
Data analysis	Student's T test was used for two-group comparisons, and one-way ANOVA with Tukey's T test was used for multiple comparisons byusing SPSS, version 26 for Windows (SPSS Inc., Chicago, IL, USA).

For manuscripts utilizing custom algorithms or software that are central to the research but not yet described in published literature, software must be made available to editors/reviewers. We strongly encourage code deposition in a community repository (e.g. GitHub). See the Nature Research guidelines for submitting code & software for further information.

Data

Policy information about availability of data

All manuscripts must include a data availability statement. This statement should provide the following information, where applicable:

- Accession codes, unique identifiers, or web links for publicly available datasets
- A list of figures that have associated raw data
- A description of any restrictions on data availability

The data that support the findings of this study are available from the corresponding author upon reasonable request.

Field-specific reporting

Please select the one below that is the best fit for your research. If you are not sure, read the appropriate sections before making your selection.

☒ Life sciences ☐ Behavioural & social sciences ☐ Ecological, evolutionary & environmental sciences

Life sciences study design

All studies must disclose on these points even when the disclosure is negative.

Sample size	No sample-size calculations were performed. Sample size was determined to be adequate based on prior literature and the magnitude and consistency of measurable differences between groups.
Data exclusions	N/A
Replication	The experiments using cell lines were replicated twice.
Randomization	Animals were randomly assigned among the various groups.
Blinding	All analyses were conducted with blinding to the experimental condition.

Reporting for specific materials, systems and methods

We require information from authors about some types of materials, experimental systems and methods used in many studies. Here, indicate whether each material, system or method listed is relevant to your study. If you are not sure if a list item applies to your research, read the appropriate section before selecting a response.

Materials & experimental systems

n/a	Involved in the study	
☐	☒	Antibodies
☐	☒	Eukaryotic cell lines
☒	☐	Palaeontology
☐	☒	Animals and other organisms
☒	☐	Human research participants
☒	☐	Clinical data

Methods

n/a	Involved in the study	
☒	☐	ChIP-seq
☐	☒	Flow cytometry
☒	☐	MRI-based neuroimaging

Antibodies

Antibodies used

anti-GAPDH antibody (Santa Cruz Biotechnology: sc-25778)
anti-PGRMC1 antibody (Cell signaling: 13856S)
anti-PPAR gamma antibody (Abcam: ab59256)
anti-FABP4 antibody (Abcam: ab66682)
anti-LDL-R antibody for western blotting (R&D: AF2255)
anti-VLDL-R antibody (R&D: AF2258)
anti-Tf-R antibody (Abcam: ab84039)
anti-Na-K ATPase alpha1 antibody (Abcam: ab7671)
anti-GLUT1 antibody (Abcam: ab115730)
anti-GLUT4 antibody (Abcam: ab33780)
anti-Akt antibody (Cell signaling: #9272S)
anti-pAkt antibody (Cell signaling: #4060S)
anti-HO-1 antibody(Santa Cruz Biotechnology: sc-136960)

Validation

anti-GAPDH antibody (Santa Cruz Biotechnology: sc-25778) was validated for detection of mouse GAPDH by WB.
anti-PGRMC1 antibody (Cell signaling: 13856S) was validated for detection of mouse PGRMC1 by WB.
anti-PPAR gamma antibody (Abcam: ab59256) was validated for detection of mouse PPAR gamma by WB.
anti-FABP4 antibody (Abcam: ab66682) was validated for detection of mouse FABP4 by WB.
anti-LDL-R antibody for western blotting (R&D: AF2255) was validated for detection of mouse LDL-R by WB.
anti-VLDL-R antibody (R&D: AF2258) was validated for detection of mouse VLDL-R by WB.
anti-Tf-R antibody (Abcam: ab84039) was validated for detection of mouse Tf-R by WB.
anti-Na-K ATPase alpha1 antibody (Abcam: ab7671) was validated for detection of mouse Na-K ATPase alpha1 by WB.
anti-GLUT1 antibody (Abcam: ab115730) was validated for detection of mouse GLUT1 by WB.
anti-GLUT4 antibody (Abcam: ab33780) was validated for detection of mouse GLUT4 by WB.
anti-Akt antibody (Cell signaling: #9272S) was validated for detection of mouse Akt by WB.
anti-pAkt antibody (Cell signaling: #4060S) was validated for detection of mouse pAkt by WB.
anti-HO-1 antibody(Santa Cruz Biotechnology: sc-136960) was validated for detection of mouse HO-1 by WB.

Eukaryotic cell lines

Policy information about cell lines

Cell line source(s)	3T3L1 cell
Authentication	None of the cell lines used were authenticated.
Mycoplasma contamination	All cell lines were not tested for mycoplasma contamination.
Commonly misidentified lines (See ICLAC register)	N/A

Animals and other organisms

Policy information about studies involving animals; ARRIVE guidelines recommended for reporting animal research

Laboratory animals	Eight-week-old C57BL/6J male mice were purchased from SLC Japan (Shizuoka, Japan). PGRMC1 exon 2 was flanked with loxP sites in C57BL/6J (WT) mice (PGRMC1 flox/flox mice). PGRMC1 flox/flox mice then were crossed with adiponectin-Cre mice, thereby generating PGRMC1 adipose tissue-specific knockout mice.
Wild animals	N/A
Field-collected samples	N/A
Ethics oversight	All the protocols for animal experiments in this study were approved by the Experimental Animal Committee of Keio University School of Medicine [approved number, 08024(10)]

Note that full information on the approval of the study protocol must also be provided in the manuscript.

Flow Cytometry

Plots

Confirm that:

☒ The axis labels state the marker and fluorochrome used (e.g. CD4-FITC).

☒ The axis scales are clearly visible. Include numbers along axes only for bottom left plot of group (a 'group' is an analysis of identical markers).

☒ All plots are contour plots with outliers or pseudocolor plots.

☒ A numerical value for number of cells or percentage (with statistics) is provided.

Methodology

Sample preparation	Cells treated with Alexa Fluor 488 acetylated LDL or DiI-VLDL were diluted in PBS containing 1% paraformaldehyde. Thaeman fluorescent intensity per 10,000 cells was analyzed using a flowcytometer (Gallious, Beckman Coulter Life Science, Brea, CA).
Instrument	Gallious
Software	Kaluza v1.5a
Cell population abundance	Cell populations were selected according to the size (forward-scatter) and complexity (side-scatter). Debris were excluded. 10,000 events per data point were collected for analysis.
Gating strategy	Cell populations were selected according to the size (forward-scatter) and complexity (side-scatter). Debris were excluded. 10,000 events per data point were collected for analysis.

☒ Tick this box to confirm that a figure exemplifying the gating strategy is provided in the Supplementary Information.
